# Genome‐wide association study of six quality traits reveals the association of the *TaRPP13L1* gene with flour colour in Chinese bread wheat

**DOI:** 10.1111/pbi.13126

**Published:** 2019-04-21

**Authors:** Jianhui Chen, Fuyan Zhang, Chunjiang Zhao, Guoguo Lv, Congwei Sun, Yubo Pan, Xinyu Guo, Feng Chen

**Affiliations:** ^1^ National Key Laboratory of Wheat and Maize Crop Science/Collaborative Innovation Center of Henan Grain Crops/Agronomy College Henan Agricultural University Zhengzhou China; ^2^ Henan Key Laboratory of Nuclear Agricultural Sciences/Isotope Institute Co., Ltd Henan Academy of Sciences Zhengzhou China; ^3^ Beijing Key Lab of Digital Plant Beijing Research Center for Information Technology in Agriculture Beijing Academy of Agriculture and Forestry Sciences Beijing China; ^4^ Beijing Research Center for Information Technology in Agriculture Beijing Academy of Agriculture and Forestry Sciences Beijing China

**Keywords:** bread wheat (*Triticum aestivum* L.), quality traits, genome‐wide association study, QTL mapping, *TaRPP13L1* gene

## Abstract

Flour colour, kernel hardness, grain protein content and wet gluten content are important quality properties that determine end use in bread wheat. Here, a wheat 90K genotyping assay was used for a genome‐wide association study (GWAS) of the six quality‐related traits in Chinese wheat cultivars in eight environments over four years. A total of 846 significant single nucleotide polymorphisms (SNPs) were identified, explaining approximately 30% of the phenotypic variation on average, and 103 multienvironment‐significant SNPs were detected in more than four environments. Quantitative trait loci (QTL) mapping in the biparent population confirmed some important SNP loci. Moreover, it was determined that some important genes were associated with the six quality traits, including some known functional genes and annotated unknown functional genes. Of the annotated unknown functional genes, it was verified that *TaRPP13L1* was associated with flour colour. Wheat cultivars or lines with *TaRPP13L1‐B1a* showed extremely significantly higher flour redness and lower yellowness than those with *TaRPP13L1‐B1b* in the Chinese wheat natural population and the doubled haploid (DH) population. Two tetraploid wheat lines with premature stop codons of the *TaRPP13L1* gene mutagenized by ethyl methanesulfonate (EMS) showed extremely significantly higher flour redness and lower yellowness than wild type. Our data suggest that the *TaRPP13L1* gene plays an important role in modulating wheat flour colour. This study provides useful information for further dissection of the genetic basis of flour colour and also provides valuable genes or genetic loci for marker‐assisted selection to improve the process of breeding quality wheat in China.

## Introduction

China is the largest producer and consumer of wheat in the world. The Yellow and Huai River Valley is the most important and largest wheat‐producing region, with 60%–70% of both total harvested area and wheat production in China (Chen *et al*., 2013). In this region, flour products are the most popular food, especially steamed bread and noodles that can be evaluated with indicators, such as kernel hardness, protein content, wet gluten content and flour colour parameters. Generally, excellent quality wheat that can be used to produce steamed bread is derived from wheat cultivars with medium–high levels of hardness, protein content and gluten content, as well as white colour. Flour with high yellowness is preferable for both Chinese and Japanese alkaline yellow noodles (Kruger *et al*., 1994), while Chinese fresh or dry white noodles and other products such as bread and dumplings require white flour with extremely low yellowness (Zhang *et al*., 2009a,b). Therefore, wheat cultivars suitable for steamed bread and noodles are preferable for growing in the Yellow and Huai River Valley. However, there is a severe shortage of the two types of wheat cultivars in China where the grain possesses all of the desired characteristics, that is cultivars with high levels of kernel hardness, protein content and wet gluten content, with suitable colour for producing bread‐type products, and cultivars with low levels of kernel hardness, protein content and wet gluten content, with suitable colour for producing cake‐type products. Therefore, improving the wheat quality to satisfy the demand for different end use has become one of the most important breeding goals in the Yellow and Huai River wheat region.

Kernel hardness, which determines international marketing classification, is a major determinant of the end use in wheat. It has been proven that kernel hardness is mainly determined by two genes, puroindoline a and b (*Pina* and *Pinb*) on 5DS, which form the molecular basis of wheat kernel hardness (Chen *et al*., 2013; Giroux and Morris, 1998). However, kernel hardness cannot be fully accounted for by *Pina* and *Pinb*. Previous studies have shown that there are many other quantitative trait loci (QTL) contributing to hardness on almost all 21 wheat chromosomes, especially on chromosomes 1B, 2A, 4B, 5A, 5B, 5D and 7D (Bordes *et al*., 2011; Li *et al*., 2016; Wang *et al*., 2012).

Grain protein content (GPC) is often used to measure the nutritional value of food. Wheat is considered as a highly nutritious crop due to the 12%–14% protein content of the grain (Shewry, 2009). Wheat protein can be divided into two major categories: prolamins, including gliadins and glutenins, and non‐prolamins, consisting of water‐soluble albumins and salt‐soluble globulins (Singh and Skerritt, 2001). Among them, the wet gluten, mainly composed of gliadins and glutenins, which comprises 60%–80% of total grain proteins, plays a key role in the unique baking quality of wheat (Torbica *et al*., 2007). The wet gluten content, which is positively correlated with protein content, is an indispensable factor in evaluating wheat quality. Usually, wheat with high protein and high wet gluten content is suitable for making bread and high‐quality noodles, and cultivars with low protein and low wet gluten content are suitable for making biscuits and cakes. Both protein and wet gluten content are controlled by multiple genes. It has been verified that the *GPC* gene on the short arm of the group 6 chromosomes regulates protein content (Uauy *et al*., 2006). Extensive QTL analysis for GPC has been performed, and many QTLs were detected on all 21 chromosomes (Bogard *et al*., 2013; Zhang *et al*., 2008a; Zhao *et al*., 2010). However, compared with the grain protein content, few studies have reported the wet gluten content (WGC), although a small amount of QTL has been detected for the WGC (Li *et al*., 2009; Sun *et al*., 2008). Furthermore, genes or QTL for gliadins or glutenins were mainly detected on the chromosomes of groups 1 and 6 (Plessis *et al*., 2013).

Consumers utilize flour colour to gauge the desirability of the end use, and it is very important in the assessment of wheat quality for many products (Parker and Langridge, 2000). The most common parameters for evaluation of flour colour are L* (lightness), a* (redness) and b* (yellowness), based on colorimeter. Theoretically, a pure white flour has zero values for a* and b*, and one hundred for L* (Zhang *et al*., 2009a). Flour colour is mainly controlled by genetic factors and polygenes. Some genes or QTLs have been isolated or identified for flour‐related traits in bread wheat by *in silico* cloning or biparent mapping, for example the phytoene synthase *(Psy1)* gene on the chromosomes of group 7, which were considered to be the most critical chromosomes affecting colour (Pozniak *et al*., 2007; Ravel *et al*., 2013; Singh *et al*., 2009), the polyphenol oxidase (*PPO*) gene on the chromosomes of group 2 (Beecher and Skinner, 2011; He *et al*., 2007; Taranto *et al*., 2015), the lipoxygenase (*LOX*) gene on the chromosomes of groups 4 and 5 (Carrera *et al*., 2007; Feng *et al*., 2012; Geng *et al*., 2012) and the lycopene ε‐cyclase (*Lcye*) gene on the chromosomes of group 3 (Crawford and Francki, 2013; Mares and Campbell, 2001). Studies on QTL for flour colour (L*, a* and b*) have been performed, and many QTLs were detected on almost all chromosomes (Kuchel *et al*., 2006; Roncallo *et al*., 2012; Zhai *et al*., 2016).

Currently, the genetic basis of quantitative variation for wheat quality traits is poorly understood. The standard QTL mapping methods only locate associated genomic regions with low resolution due to limited marker numbers and genetic background for biparental population (Sukumaran *et al*., 2015). Recently, with the rapid development of next‐generation sequencing (NGS) and high‐density marker genotyping techniques, genome‐wide association study (GWAS) has become an efficient method to identify the genetic basis for the quantitative variation of complex traits in crop plants (Nordborg and Weigel, 2008; Zhu *et al*., 2008). GWAS has been frequently used to examine agronomic traits in wheat (Liu *et al*., 2017; Mengistu *et al*., 2016; Sukumaran *et al*., 2015; Sun *et al*., 2017). However, because of the requirement for large amounts of seeds and the long‐time determination of some quality traits, few GWASs of the natural wheat population have been performed, thus far for wheat quality traits.

In this study, we performed a GWAS for six important quality traits in eight environments with the aim of identifying some environmentally stable new significant SNP loci and candidate genes in the entire genome of bread wheat. This study could provide valuable information to further reveal the genetic basis of the six important quality traits and provide guidance for further marker‐assisted selection of wheat in the wheat breeding programme of the Yellow and Huai River Valley of China.

## Results

### Phenotypic variation

**Figure 1:**
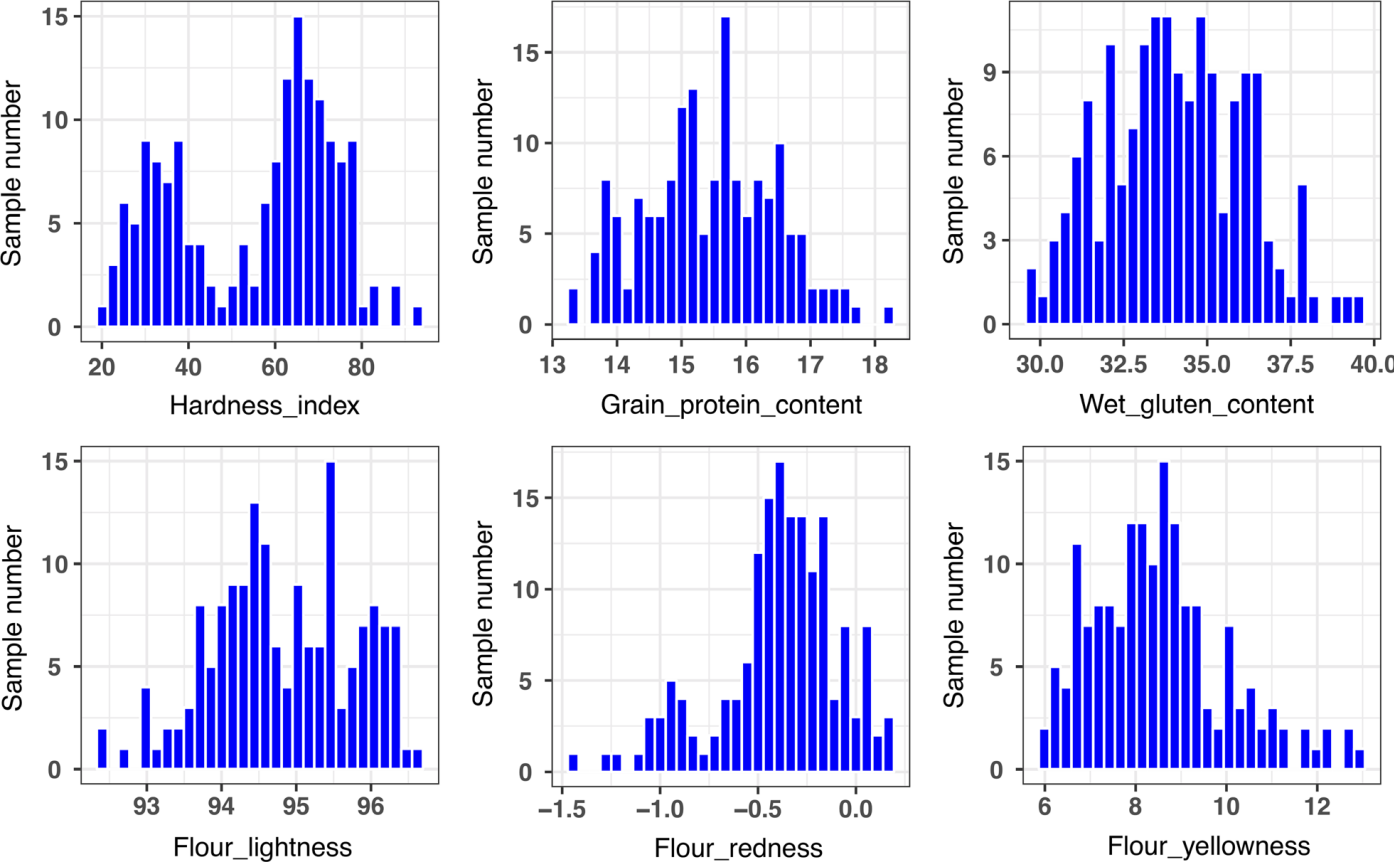
Phenotype distribution of six quality traits in the associated population.

The grain protein content (GPC), wet gluten content (WGC), flour lightness (FL*), flour redness (Fa*) and flour yellowness (Fb*) were nearly symmetrically distributed in the cultivars surveyed (Figures 1, S1). However, there was a bipolar distribution for the hardness index (HI; Figures 1, S1), suggesting that hard wheat is prevalent in the cultivars from the Yellow and Huai River wheat region, followed by soft wheat. Based on the averaged values over all eight environments, analysis of Pearson's coefficients of correlation among six investigated traits (Table S1A) showed that the HI was significantly and positively correlated with Fb* (*r *=* *0.590**), but was significantly and negatively correlated with FL* (*r *=* *−0.834**). GPC was significantly and positively correlated with WGC (*r *=* *0.895**) and Fa* (*r *=* *0.306**). GPC, WGC and Fa* were significantly and positively correlated between any two of the three traits (*r *=* *0.895** between GPC and WGC, *r *=* *0.306** between GPC and Fa*, and *r *=* *0.319** between WGC and Fa*). Among three flour colour parameters, Fb* was significantly and negatively correlated with FL* (*r *=* *−0.638**) and Fa* (*r *=* *−0.808**). Coefficients of correlation among eight environments showed that the six quality traits possessed stable phenotypes (Table S1B).

### Genome‐wide association study

**Figure 2:**
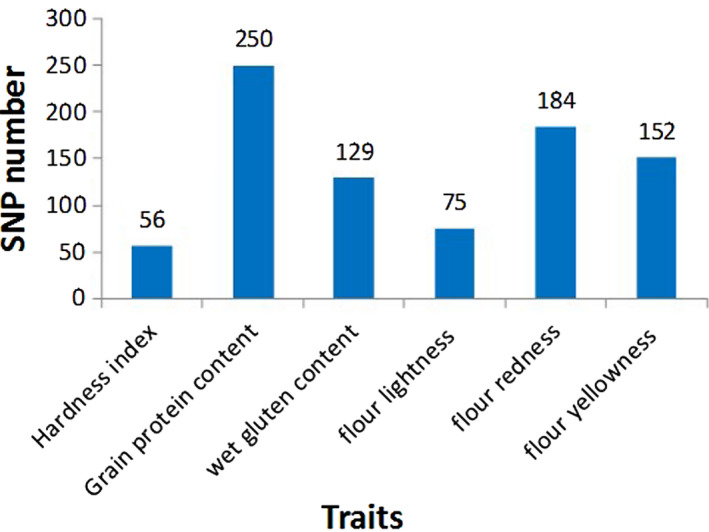
The number of associated significant SNPs for six quality traits.

After filtering, 44 790 SNPs were retained for the GWAS. Association mapping of these SNPs with the six traits (i.e. HI, GPC, WGC, FL*, Fa* and Fb*) identified 846 significant SNPs (*P* < 1.0e‐3) in the eight environments (Figure 2). Of them, 103 SNPs were significant in more than four environments. Based on the multienvironment significance of SNPs and phenotypic variation explained (PVE), 58 important SNPs that were significant in more than five environments are summarized in Table 1.

**Table 1:** Multienvironment‐significant SNPs and their *P* values identified in more than five of the investigated environments and the candidate genes

Traits	SNP	Chromosome	Position	E1	E2	E3	E4	E5	E6	E7	E8	Candidate gene
HI	Kukri_c34553_89	2BL	8075586	9.06E‐04	8.11E‐04	2.47E‐04	ns	ns	4.98E‐04	8.45E‐04	ns	*STK*
	wsnp_Ku_c4078_7436510	3B	10719102	6.12E‐04	9.12E‐05	8.98E‐04	4.45E‐04	5.57E‐04	ns	ns	ns	
	IAAV7827	5AL	1451825	2.42E‐04	1.03E‐04	1.09E‐04	5.02E‐05	1.61E‐04	1.65E‐04	9.51E‐05	1.67E‐04	
	Kukri_rep_c102608_599	5AL	2672454	6.40E‐04	5.71E‐05	1.27E‐04	9.70E‐05	8.74E‐04	2.60E‐04	4.96E‐04	6.88E‐04	
	BS00000020_51	5DS	2781261	3.89E‐12	4.85E‐11	2.07E‐10	1.26E‐12	4.74E‐12	5.65E‐12	3.25E‐13	4.03E‐13	*Pinb*
GPC	RFL_Contig2972_2819	2BL	8026587	2.45E‐05	6.93E‐04	7.17E‐04	8.02E‐04	ns	6.58E‐04	ns	9.11E‐05	
	RAC875_c3187_873	2DL	9879600	9.26E‐04	ns	ns	4.49E‐05	3.31E‐04	2.68E‐04	9.18E‐04	3.63E‐05	
	BS00046964_51	6AS	4416291	3.68E‐04	7.52E‐04	3.54E‐04	1.97E‐05	5.58E‐05	4.52E‐04	9.80E‐05	2.08E‐05	*RPL1*
	IAAV5188	6AS	4386834	8.15E‐04	ns	6.30E‐04	5.31E‐05	8.54E‐05	ns	3.25E‐04	5.77E‐05	
	Ra_c28284_223	6AS	4389186	ns	5.49E‐04	3.37E‐04	1.48E‐04	6.32E‐04	ns	2.12E‐04	1.04E‐04	
	Tdurum_contig51717_1582	6AS	4429229	ns	8.89E‐04	5.02E‐04	1.36E‐04	7.65E‐04	ns	3.12E‐04	1.29E‐04	
	BS00010811_51	6AS	4429229	ns	8.89E‐04	5.02E‐04	1.36E‐04	7.65E‐04	ns	3.12E‐04	1.29E‐04	
	wsnp_Ex_c24958_34212226	6AS	4351123	ns	ns	9.00E‐04	9.72E‐05	4.30E‐04	ns	2.77E‐04	1.33E‐04	*SPY*
	wsnp_Ex_c43412_49738738	6AS	4351123	ns	ns	9.00E‐04	9.72E‐05	4.30E‐04	ns	2.77E‐04	1.33E‐04	*SPY*
	BS00010441_51	6DS	1696321	ns	ns	9.00E‐04	9.72E‐05	4.30E‐04	ns	2.77E‐04	1.33E‐04	
	BS00043554_51	7BL	6738036	6.95E‐04	3.19E‐04	7.79E‐04	ns	ns	ns	4.51E‐04	2.50E‐04	
WGC	RAC875_c3187_873	2DL	9879600	8.88E‐04	ns	8.66E‐04	3.36E‐04	8.65E‐04	ns	ns	1.34E‐04	
	BS00046964_51	6AS	4416291	ns	ns	ns	1.99E‐04	9.24E‐05	2.83E‐04	5.76E‐04	1.65E‐04	*RPL1*
	IAAV5188	6AS	4386834	ns	ns	ns	5.21E‐04	1.36E‐04	5.85E‐04	9.94E‐04	4.75E‐04	
FL*	BS00029347_51	5AS	1535523	5.88E‐04	ns	3.71E‐04	3.36E‐04	6.31E‐04	5.02E‐04	3.83E‐04	5.49E‐04	
	BS00041219_51	5AS	1535523	8.87E‐04	ns	4.92E‐04	3.53E‐04	8.55E‐04	4.52E‐04	3.58E‐04	6.59E‐04	
	wsnp_Ra_c24707_34262900	5AS	1535523	8.87E‐04	ns	4.92E‐04	3.53E‐04	8.55E‐04	4.52E‐04	3.58E‐04	6.59E‐04	
	IAAV7827	5AL	1451825	ns	ns	ns	2.43E‐05	4.15E‐04	2.21E‐04	1.18E‐04	1.34E‐04	
	BS00000020_51	5DS	2781261	1.38E‐06	4.42E‐07	9.61E‐07	1.71E‐08	9.71E‐08	6.11E‐08	8.86E‐09	4.57E‐08	*Pinb*
Fa*	Ex_c16529_304	1AL	2752572	1.06E‐04	1.46E‐04	6.51E‐04	1.11E‐04	2.53E‐04	ns	4.69E‐05	ns	*MCM3*
	Excalibur_c42979_666	2AL	6406239	2.90E‐08	3.05E‐07	6.46E‐08	1.77E‐06	1.54E‐04	5.80E‐07	1.13E‐05	8.35E‐06	
	BS00104177_51	3B	10664131	1.25E‐04	2.12E‐04	5.19E‐04	5.50E‐04	ns	6.35E‐04	5.25E‐04	2.89E‐04	
	Kukri_rep_c110544_52	3B	10457438	1.25E‐04	2.12E‐04	5.19E‐04	5.50E‐04	ns	6.35E‐04	5.25E‐04	2.89E‐04	
	Excalibur_c20309_539	3B	10664131	1.25E‐04	2.12E‐04	5.19E‐04	5.50E‐04	ns	6.35E‐04	5.25E‐04	2.89E‐04	
	BS00003703_51	7AL	4478034	2.00E‐06	3.43E‐06	1.24E‐06	1.04E‐04	ns	7.66E‐05	6.97E‐04	9.54E‐04	*Psy‐7A*
	RFL_Contig2982_586	7AL	4536617	1.27E‐05	5.04E‐05	2.41E‐05	8.29E‐04	ns	1.58E‐04	ns	ns	
	tplb0026f23_1134	7BL	6691403	4.33E‐07	9.63E‐06	1.46E‐06	7.22E‐05	ns	2.84E‐05	1.12E‐04	6.37E‐05	*Psy‐7B*
	Excalibur_c8883_1144	7BL	6751305	3.82E‐07	2.43E‐06	1.97E‐06	9.97E‐06	1.94E‐04	3.61E‐06	7.49E‐04	ns	
	Excalibur_c5938_1846	7BL	6748067	1.27E‐06	8.94E‐06	2.53E‐06	6.42E‐05	ns	5.57E‐05	3.53E‐04	2.89E‐04	*RPP13L1‐7B*
	Excalibur_c5938_1703	7BL	6748067	1.27E‐06	8.94E‐06	2.53E‐06	6.42E‐05	ns	5.57E‐05	3.53E‐04	2.89E‐04	*RPP13L1‐7B*
	BobWhite_c10975_60	7BL	6748067	1.27E‐06	8.94E‐06	2.53E‐06	6.42E‐05	ns	5.57E‐05	3.53E‐04	2.89E‐04	*RPP13L1‐7B*
	Kukri_c4352_194	7BL	6752037	2.90E‐08	3.05E‐07	6.46E‐08	1.77E‐06	1.54E‐04	5.80E‐07	1.13E‐05	8.35E‐06	
	RAC875_c38693_499	7BL	6752037	2.90E‐08	3.05E‐07	6.46E‐08	1.77E‐06	1.54E‐04	5.80E‐07	1.13E‐05	8.35E‐06	
	Excalibur_c5851_1661	7BL	6741777	2.23E‐04	ns	7.29E‐04	6.65E‐05	3.09E‐04	2.27E‐04	2.18E‐04	1.54E‐04	
	Kukri_c65663_642	7DL	3295682	8.28E‐07	4.96E‐06	1.13E‐06	4.10E‐05	6.15E‐04	2.09E‐05	4.55E‐04	7.14E‐04	*RPP13L1‐7D*
	RAC875_c14064_308	7DL	3295682	2.18E‐04	5.11E‐04	6.94E‐04	3.55E‐05	7.27E‐04	2.31E‐04	ns	ns	*RPP13L1‐7D*
	Excalibur_c8883_214	7DL	3364136	3.13E‐05	2.58E‐04	6.31E‐05	ns	ns	1.64E‐04	7.95E‐05	1.83E‐04	
	RAC875_c61016_73	7DL	3393253	7.46E‐06	7.71E‐05	2.65E‐05	5.09E‐04	ns	4.02E‐05	ns	ns	
	Excalibur_rep_c92684_578	7DL	3391573	4.48E‐08	3.44E‐07	1.43E‐07	1.00E‐06	1.51E‐05	2.09E‐06	2.09E‐06	3.64E‐06	
Fb*	Ex_c16529_304	1AL	2752572	ns	ns	ns	1.59E‐04	1.07E‐04	2.64E‐04	3.93E‐04	5.81E‐04	*MCM3*
	Excalibur_c42979_666	2AL	6406239	6.71E‐06	4.60E‐04	1.61E‐04	3.27E‐04	6.47E‐04	7.22E‐04	2.51E‐04	2.80E‐04	
	RAC875_rep_c117294_342	3B	10677569	1.53E‐04	1.16E‐04	4.32E‐05	7.15E‐04	ns	ns	ns	9.00E‐04	
	RAC875_rep_c118396_333	3B	10677569	1.53E‐04	1.16E‐04	4.32E‐05	7.15E‐04	ns	ns	ns	9.00E‐04	
	BS00104177_51	3B	10664131	1.84E‐04	6.26E‐04	9.94E‐04	7.53E‐04	6.24E‐04	1.84E‐04	7.71E‐04	4.97E‐04	
	Kukri_rep_c110544_52	3B	10457438	1.84E‐04	6.26E‐04	9.94E‐04	7.53E‐04	6.24E‐04	1.84E‐04	7.71E‐04	4.97E‐04	
	Excalibur_c20309_539	3B	10664131	1.84E‐04	6.26E‐04	9.94E‐04	7.53E‐04	6.24E‐04	1.84E‐04	7.71E‐04	4.97E‐04	
	BS00000020_51	5DS	2781261	1.21E‐06	1.21E‐06	2.73E‐06	1.13E‐07	3.86E‐07	2.39E‐06	9.86E‐08	1.71E‐07	*Pinb*
	Kukri_rep_c68371_1242	7AL	4552612	4.09E‐04	8.74E‐05	ns	7.39E‐04	ns	5.34E‐04	ns	6.79E‐04	*SBE1*
	tplb0026f23_1134	7BL	6691403	9.57E‐06	3.86E‐04	1.53E‐04	9.67E‐04	9.12E‐04	ns	ns	9.19E‐04	*Psy‐7B*
	Kukri_c4352_194	7BL	6752037	6.71E‐06	4.60E‐04	1.61E‐04	3.27E‐04	6.47E‐04	7.22E‐04	2.51E‐04	2.80E‐04	
	RAC875_c38693_499	7BL	6752037	6.71E‐06	4.60E‐04	1.61E‐04	3.27E‐04	6.47E‐04	7.22E‐04	2.51E‐04	2.80E‐04	
	Excalibur_c5851_1661	7BL	6741777	8.53E‐04	ns	5.83E‐04	7.56E‐04	5.63E‐04	9.22E‐04	ns	8.68E‐04	
	Excalibur_rep_c92684_578	7DL	3391573	4.28E‐06	1.29E‐04	2.04E‐05	4.28E‐05	9.79E‐05	4.48E‐05	9.56E‐05	4.94E‐05	

Fa*, flour redness; Fb*, flour yellowness; FL*, flour lightness; GPC, grain protein content; HI, hardness index; *MCM3,* DNA replication licensing factor MCM3 gene; *Pinb*, puroindoline‐b gene; *Psy,* phytoene synthase gene; *RPL1,* 50S ribosomal protein L3‐1, chloroplastic gene; *RPP13L1,* RPP13‐like protein 1 gene; *SBE1,* starch‐branching enzyme I gene; *SPY,* UDP‐N‐acetylglucosamine‐peptide N‐acetylglucosaminyltransferase SPINDLY gene; *STK*, serine/threonine‐protein kinase gene; WGC, wet gluten content. With the exception that the *RPP13L1* gene was predicted, other genes were annotated.

E1, 2013 Anyang; E2, 2013 Zhengzhou; E3, 2013 Zhumadian. E4, 2014 Anyang; E5, 2014 Zhengzhou; E6, 2014 Zhumadian; E7, 2015 Zhengzhou; E8, 2016 Zhengzhou.

### Hardness index

**Figure 3:**
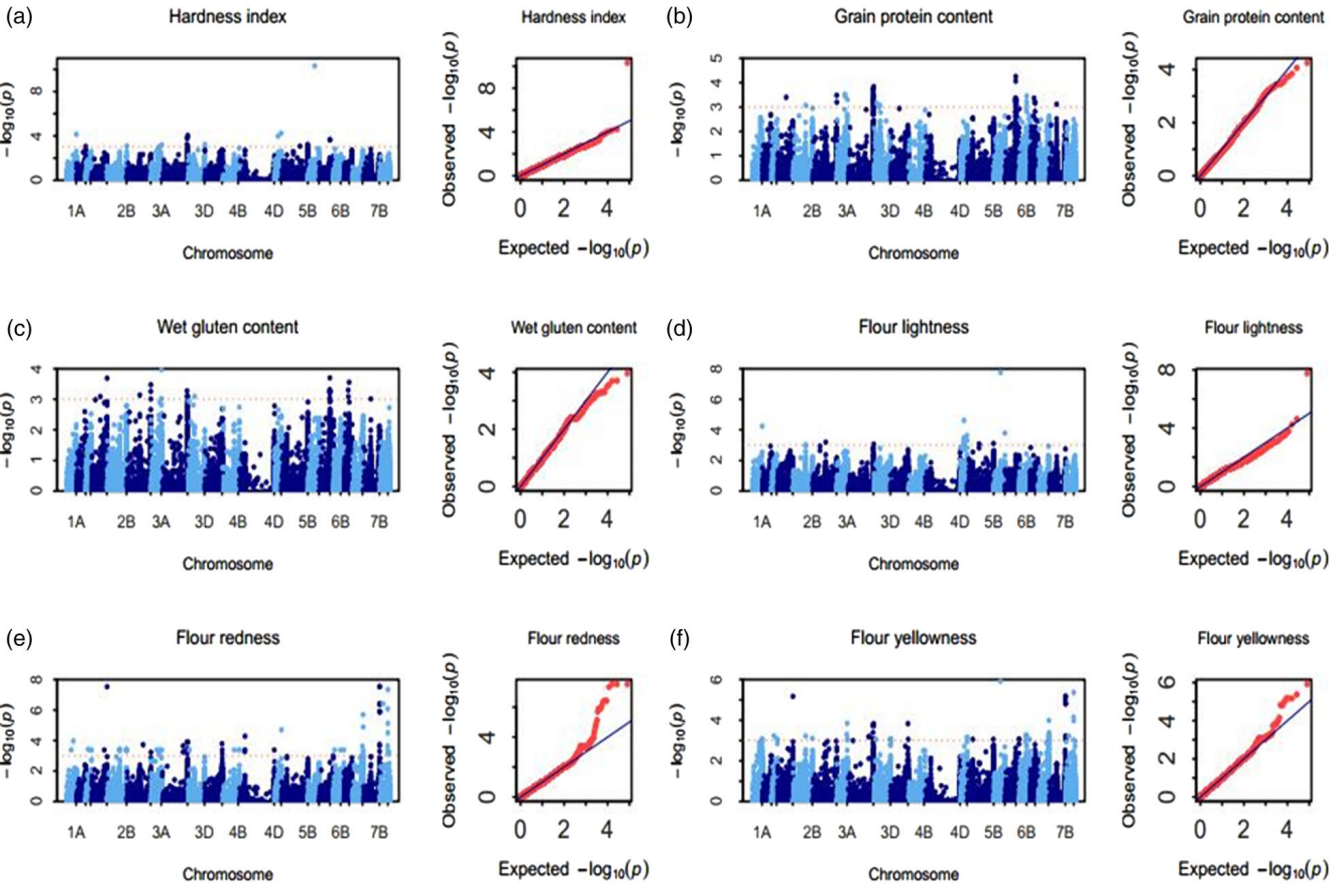
Manhattan and Q–Q plots for six quality traits. (a) Hardness index (HI), (b) grain protein content (GPC), (c) wet gluten content (WGC), (d) flour lightness (FL*), (e) flour redness (Fa*) and (f) flour yellowness (Fb*).

Fifty‐six SNPs, mainly distributing on chromosomes 1AL, 2BL, 3B, 5AL, 5BL, 5DS and 7AL, were significantly associated with the hardness index (Tables 1, S2; Figures 3a, S2). The PVE of each SNP ranged from 11.3% to 45.3%. Of all of the multienvironment‐significant SNPs, the most significant SNP BS00000020_51 on 5DS (with PVE of 40.9%) was detected in all 8 environments and showed a negative effect to the hardness index. On 5AL, IAAV7827 with PVE of 17.2% and Kukri_rep_c102608_599 with PVE of 16.3% were both detected in all 8 environments. IAAV7827 showed a negative effect on the HI, while Kukri_rep_c102608_599 exhibited a positive effect on the HI. Kukri_c34553_89 with PVE of 15.8% on 2BL and wsnp_Ku_c4078_7436510 with PVE of 15.6% on 3B were significant in 5 environments, and Kukri_c34553_89 showed a negative effect on the HI, but wsnp_Ku_c4078_7436510 exhibited a positive effect on HI. Because wheat kernel hardness is mainly affected by the *Pina* and *Pinb* genes, mutation of *Pina* or *Pinb* causes a hard endosperm. Based on the blast analysis of whole‐genome sequences of Chinese Spring and AK58, BS00000020_51 was determined to be part of the sequence of the *Pinb* gene (Table 1). Additionally, the other four SNPs (Kukri_c34553_89, wsnp_Ku_c4078_7436510, IAAV7827 and Kukri_rep_c102608_599) all have greater impact in soft wheat than in hard wheat (Table S2).

### Grain protein content

Two hundred and fifty SNPs, mainly distributing on chromosomes 2BL, 2DL, 6AS, 6BL, 6DS and 7BL, were significantly associated with GPC (Tables 1, S3; Figures 3b, S3). The PVE of each SNP ranged from 13.9% to 32.1%. Of all the significant SNPs, 7 multienvironment‐significant SNPs on 6AS (BS00046964_51, IAAV5188, Ra_c28284_223, Tdurum_contig51717_1582, BS00010811_51, wsnp_Ex_c24958_34212226 and wsnp_Ex_c43412_49738738) were significantly associated with GPC in 8, 6, 6, 6, 6, 5 and 5 environments with an averaged PVE of 23.7%, 23.6%, 23.9%, 23.6%, 23.6%, 22.8% and 22.8%, respectively. Of the 7 multienvironment‐significant SNPs, BS00046964_51, Ra_c28284_223, Tdurum_contig51717_1582 and wsnp_Ex_c24958_34212226 showed a positive effect to GPC, while IAAV5188, BS00010811_51 and wsnp_Ex_c43412_49738738 displayed a negative effect to GPC. In addition, RFL_Contig2972_2819 (with PVE of 21.7%) on 2BL, RAC875_c3187_873 (with PVE of 22.0%) on 2DL, BS00010441_51 (with PVE of 22.9%) on 6DS and BS00043554_51 (with PVE of 26.4%) on 7BL were also detected in 6, 6, 5 and 5 environments, respectively. Of them, RFL_Contig2972_2819, RAC875_c3187_873 and BS00043554_51 showed a negative effect to protein content, while BS00010441_51 displayed a positive effect to protein content.

### Wet gluten content

One hundred and twenty‐nine SNPs, mainly located on chromosomes 2DL, 3AL and 6AS, were significantly associated with WGC, and the PVE of each SNP ranged from 10.4% to 33.3% (Tables 1, S4; Figures 3c, S4). Of these SNPs, BS00046964_51 (with PVE of 23.8%) and IAAV5188 (with PVE of 23.0%) on 6AS were significantly associated with WGC in the same 5 environments, and BS00046964_51 showed a positive effect to WGC but IAAV5188 displayed a negative effect to WGC. Meanwhile, RAC875_c3187_873 (with PVE of 19.6%) on 2DL was significantly and negatively associated with WGC in 5 environments.

### Flour colour parameters

#### Flour lightness (FL*)

Seventy‐five SNPs, mainly distributing on chromosomes 1AL, 5AS, 5AL, 5BL, 5DS and 6AS, were significantly associated with FL*, and PVE of each SNP ranged from 12.7% to 29.0% (Tables 1, S5; Figures 3d, S5). Of all significant SNPs, BS00000020_51 on 5DS (i.e. *Pinb* as mentioned above) was most significantly associated with FL* in all 8 environments, which explained the 26.6% phenotypic variation on average and showed a negative effect to FL*. On 5AS, BS00029347_51 (with PVE of 15.3%), BS00041219_51 (with PVE of 15.1%) and wsnp_Ra_c24707_34262900 (with PVE of 15.1%) were all significantly associated with FL* in the same 7 environments, and BS00029347_51 and BS00041219_51 showed a positive effect to FL*, while wsnp_Ra_c24707_34262900 exhibited a negative effect to FL*. Additionally, IAAV7827 (with PVE of 15.6%) on 5AL was detected in 5 environments and showed a positive effect to FL*.

#### Flour redness (Fa*)

One hundred and eighty‐four SNPs, mainly distributing on chromosomes 1AL, 1DL, 2AL, 2DL, 3B, 4AL, 4DS, 7AL, 7BL and 7DL, were significantly associated with Fa*, and the PVE of each SNP ranged from 44.0% to 58.9% (Tables 1, S6; Figures 3e, S6). Of all of the significant SNPs, Ex_c16529_304 (with PVE of 50.3%) on 1AL and Excalibur_c42979_666 (with PVE of 54.9%) on 2AL were significant in 6 and 8 environments, respectively, and both of them showed a positive effect to Fa*. On 3B, BS00104177_51, Kukri_rep_c110544_52 and Excalibur_c20309_539 were all detected to explain an average PVE of 50.4% in the same 7 environments, and BS00104177_51 showed a positive effect to Fa*, while Kukri_rep_c110544_52 and Excalibur_c20309_539 exhibited a negative effect to Fa*. BS00003703_51 (with PVE of 52.3%) and RFL_Contig2982_586 (with PVE of 50.6%) on 7AL were significantly associated with Fa* in 7 and 5 environments, respectively. RFL_Contig2982_586 showed a positive effect to Fa*, while BS00003703_51 displays a negative effect to Fa*. On 7BL, Kukri_c4352_194 (with PVE of 54.9%), RAC875_c38693_499 (with PVE of 54.9%), Excalibur_c5851_1661 (with PVE of 51.7%), tplb0026f23_1134 (with PVE of 52.9%), Excalibur_c5938_1846 (with PVE of 52.4%), BobWhite_c10975_60 (with PVE of 52.4%), Excalibur_c5938_1703 (with PVE of 52.4%) and Excalibur_c8883_1144 (with PVE of 53.0%) were significantly associated with Fa* in 8, 8, 7, 7, 7, 7, 7 and 7 environments, respectively, and four (RAC875_c38693_499, Excalibur_c5851_1661, BobWhite_c10975_60 and Excalibur_c5938_1703) of them showed a positive effect to Fa*, while the remaining four SNPs (Kukri_c4352_194, tplb0026f23_1134, Excalibur_c5938_1846 and Excalibur_c8883_1144) exhibited a negative effect to Fa*. On 7DL, Excalibur_rep_c92684_578 (with PVE of 55.1%), Kukri_c65663_642 (with PVE of 52.5%), Excalibur_c8883_214 (with PVE of 50.1%), RAC875_c14064_308 (with PVE of 50.0%) and RAC875_c61016_73 (with PVE of 50.9%) were significantly associated with Fa* in 8, 8, 6, 6 and 5 environments, respectively, and three (Kukri_c65663_642, RAC875_c14064_308 and RAC875_c61016_73) of them showed a positive effect to Fa*, while two (Excalibur_rep_c92684_578 and Excalibur_c8883_214) of them exhibited a negative effect to Fa*. Based on blast analysis of the entire genome sequences of Chinese Spring and AK58, BS00003703_51 on 7A and tplb0026f23_1134 on 7BL are annotated as homologous phytoene synthase (*Psy*) genes (Table 1), which have been confirmed to be associated with flour colour in previous studies (Zhang *et al*., 2008a).

#### Flour yellowness (Fb*)

One hundred and fifty‐two SNPs, mainly distributing on chromosomes 1AL, 2AS, 2AL, 2BL, 3B, 4AL, 5BL, 5DS, 6BS, 7AL, 7BL and 7DL, were significantly associated with Fb*, and the PVE of each SNP ranged from 32.6% to 52.1% (Tables 1, S7; Figures 3f, S7). Of all of the significant SNPs, Ex_c16529_304 (with PVE of 42.2%) on 1AL was detected in 5 environments and showed a negative effect to Fb*. Excalibur_c42979_666 (with PVE of 40.2%) on 2AL was significantly associated with Fb* in all 8 environments and showed a negative effect to Fb*. On 3B, RAC875_rep_c117294_342 (with PVE of 39.4%), RAC875_rep_c118396_333 (with PVE of 39.4%), BS00104177_51 (with PVE of 39.5%), Kukri_rep_c110544_52 (with PVE of 39.5%) and Excalibur_c20309_539 (with PVE of 39.5%) were significantly associated with Fb* in 5, 5, 8, 8 and 8 environments, respectively, and three (RAC875_rep_c117294_342, RAC875_rep_c118396_333 and BS00104177_51) of them showed a positive effect to Fb*, while two (Kukri_rep_c110544_52 and Excalibur_c20309_539) of them exhibited a negative effect to Fb*. BS00000020_51 (i.e. *Pinb* gene) on 5DS (with PVE of 45.5%), which was the most significant SNP with Fb*, was identified in all 8 environments and showed a positive effect to Fb*. On 7AL, Kukri_rep_c68371_1242 (with PVE of 39.4%) was significantly associated with Fb* in 5 environments and showed a positive effect to Fb*. On 7BL, Kukri_c4352_194 (with PVE of 40.2%), RAC875_c38693_499 (with PVE of 40.2%), Excalibur_c5851_1661 (with PVE of 39.2%) and tplb0026f23_1134 (i.e. *Psy‐7B* with PVE of 39.3%) were significantly associated with Fb* in 8, 7, 6 and 5 environments, respectively. Of them, Kukri_c4352_194 and tplb0026f23_1134 showed a positive effect to Fb*, while RAC875_c38693_499 and Excalibur_c5851_1661 exhibited a negative effect to Fb*. On 7DL, Excalibur_rep_c92684_578 (with PVE of 41.6%) was significantly associated with Fb* in all 8 environments and showed a positive effect to Fb*.

### Validation of multienvironment‐significant SNPs in the natural population

The 58 multienvironment‐significant SNPs were verified to be significantly associated with the 6 quality traits in the natural population by *t*‐test (Table 2), and the differences in phenotypic values between cultivars with two alleles at each of these SNP loci reached extremely significant levels (*P *<* *0.01).

**Table 2:** *P* values of *t*‐test for multienvironment‐significant SNPs in different quality traits

Traits	SNP	Chromosome	Allele		Number		Phenotype		E1	E2	E3	E4	E5	E6	E7	E8
HI	Kukri_c34553_89	2BL	CC	TC	84	30	58.3	46.9	0.001	0.000	0.001	0.000	0.005	0.001	0.002	0.002
	wsnp_Ku_c4078_7436510	3B	TC	CC	60	103	60.6	50.3	0.000	0.000	0.000	0.000	0.000	0.000	0.001	0.000
	IAAV7827	5AL	AA	GG	106	53	59.2	44.8	0.000	0.000	0.000	0.000	0.000	0.000	0.000	0.000
	Kukri_rep_c102608_599	5AL	TC	CC	87	74	59.7	47.7	0.000	0.000	0.000	0.000	0.000	0.000	0.000	0.000
	BS00000020_51	5DS	TT	CC	81	71	64.5	42.1	0.000	0.000	0.000	0.000	0.000	0.000	0.000	0.000
GPC	RFL_Contig2972_2819	2BL	GG	AG	118	39	13.7	13.2	0.000	0.000	0.000	0.001	0.007	0.008	0.444	0.001
	RAC875_c3187_873	2DL	CT	TT	37	126	13.8	13.4	0.014	0.039	0.060	0.002	0.007	0.016	0.003	0.004
	BS00046964_51	6AS	GG	AG	82	77	13.8	13.2	0.000	0.000	0.000	0.000	0.000	0.000	0.000	0.000
	IAAV5188	6AS	AG	GG	82	79	13.8	13.2	0.000	0.000	0.000	0.000	0.000	0.000	0.000	0.000
	Ra_c28284_223	6AS	GG	AG	80	81	13.8	13.2	0.000	0.000	0.000	0.000	0.000	0.000	0.000	0.000
	Tdurum_contig51717_1582	6AS	GG	AA	79	81	13.8	13.2	0.000	0.000	0.000	0.000	0.000	0.000	0.000	0.000
	BS00010811_51	6AS	CC	TT	78	81	13.8	13.2	0.000	0.000	0.000	0.000	0.000	0.000	0.000	0.000
	wsnp_Ex_c24958_34212226	6AS	CC	AC	81	79	13.8	13.2	0.000	0.000	0.000	0.000	0.000	0.000	0.000	0.000
	wsnp_Ex_c43412_49738738	6AS	AA	GA	81	79	13.8	13.2	0.000	0.000	0.000	0.000	0.000	0.000	0.000	0.000
	BS00010441_51	6DS	GG	AG	81	79	13.8	13.2	0.000	0.000	0.000	0.000	0.000	0.000	0.000	0.000
	BS00043554_51	7BL	AA	GA	118	42	13.7	13.1	0.000	0.001	0.000	0.015	0.001	0.000	0.000	0.000
WGC	RAC875_c3187_873	2DL	CT	TT	37	126	30.4	29.5	0.004	0.031	0.005	0.007	0.002	0.009	0.011	0.002
	BS00046964_51	6AS	GG	AG	82	77	30.3	29.1	0.000	0.000	0.000	0.000	0.000	0.000	0.000	0.000
	IAAV5188	6AS	AG	GG	82	79	30.3	29.1	0.000	0.001	0.001	0.000	0.000	0.000	0.000	0.000
FL*	BS00029347_51	5AS	GG	AA	110	48	95.4	94.9	0.001	0.013	0.001	0.000	0.000	0.000	0.000	0.000
	BS00041219_51	5AS	GG	AG	109	37	95.4	95.0	0.008	0.103	0.027	0.004	0.002	0.006	0.003	0.003
	wsnp_Ra_c24707_34262900	5AS	CC	TC	109	36	95.4	95.0	0.008	0.105	0.034	0.006	0.003	0.006	0.003	0.003
	IAAV7827	5AL	GG	AA	53	106	95.7	95.1	0.000	0.001	0.000	0.000	0.000	0.000	0.000	0.000
	BS00000020_51	5DS	CC	TT	71	81	95.7	94.9	0.000	0.000	0.000	0.000	0.000	0.000	0.000	0.000
Fa*	Ex_c16529_304	1AL	TT	CC	124	24	−0.46	−0.72	0.000	0.000	0.000	0.000	0.000	0.000	0.000	0.002
	Excalibur_c42979_666	2AL	GA	AA	136	21	−0.44	−0.97	0.000	0.000	0.000	0.000	0.000	0.000	0.000	0.000
	BS00104177_51	3B	CT	TT	128	34	−0.47	−0.69	0.000	0.000	0.000	0.000	0.000	0.001	0.001	0.001
	Kukri_rep_c110544_52	3B	TT	CT	128	34	−0.47	−0.69	0.000	0.000	0.000	0.000	0.000	0.001	0.001	0.001
	Excalibur_c20309_539	3B	TT	CT	128	34	−0.47	−0.69	0.000	0.000	0.000	0.000	0.000	0.001	0.001	0.001
	BS00003703_51	7AL	CT	TT	97	24	−0.42	−0.92	0.000	0.000	0.000	0.000	0.000	0.000	0.000	0.000
	RFL_Contig2982_586	7AL	AG	GG	153	10	−0.48	−0.99	0.000	0.000	0.000	0.000	0.000	0.000	0.000	0.000
	tplb0026f23_1134	7BL	AA	GG	110	23	−0.46	−0.94	0.000	0.000	0.000	0.000	0.000	0.000	0.000	0.000
	Excalibur_c8883_1144	7BL	CT	TT	109	20	−0.45	−0.96	0.000	0.000	0.000	0.000	0.000	0.000	0.000	0.000
	Excalibur_c5938_1846	7BL	AA	GA	115	22	−0.44	−0.95	0.000	0.000	0.000	0.000	0.000	0.000	0.000	0.000
	Excalibur_c5938_1703	7BL	TG	GG	138	25	−0.44	−0.91	0.000	0.000	0.000	0.000	0.000	0.000	0.000	0.000
	BobWhite_c10975_60	7BL	CT	TT	111	23	−0.44	−0.93	0.000	0.000	0.000	0.000	0.000	0.000	0.000	0.000
	Kukri_c4352_194	7BL	AA	GA	141	22	−0.44	−0.96	0.000	0.000	0.000	0.000	0.000	0.000	0.000	0.000
	RAC875_c38693_499	7BL	TT	CT	139	22	−0.44	−0.96	0.000	0.000	0.000	0.000	0.000	0.000	0.000	0.000
	Excalibur_c5851_1661	7BL	TT	CT	134	17	−0.43	−0.95	0.000	0.000	0.000	0.000	0.000	0.000	0.000	0.000
	Kukri_c65663_642	7DL	GA	AA	112	23	−0.44	−0.93	0.000	0.000	0.000	0.000	0.000	0.000	0.000	0.000
	RAC875_c14064_308	7DL	TC	CC	117	39	−0.43	−0.76	0.000	0.000	0.000	0.000	0.000	0.000	0.000	0.000
	Excalibur_c8883_214	7DL	AA	GA	146	17	−0.46	−0.95	0.000	0.000	0.000	0.000	0.000	0.000	0.000	0.000
	RAC875_c61016_73	7DL	TT	CT	136	24	−0.44	−0.89	0.000	0.000	0.000	0.000	0.000	0.000	0.000	0.000
	Excalibur_rep_c92684_578	7DL	AA	GA	136	22	−0.44	−0.96	0.000	0.000	0.000	0.000	0.000	0.000	0.000	0.000
Fb*	Ex_c16529_304	1AL	TT	CC	124	24	8.38	9.76	0.000	0.000	0.000	0.000	0.000	0.000	0.000	0.000
	Excalibur_c42979_666	2AL	GA	AA	136	21	8.36	10.56	0.000	0.000	0.000	0.000	0.000	0.000	0.000	0.000
	RAC875_rep_c117294_342	3B	CT	TT	118	41	8.42	9.41	0.000	0.000	0.000	0.000	0.001	0.002	0.000	0.000
	RAC875_rep_c118396_333	3B	AC	CC	118	41	8.41	9.41	0.000	0.000	0.000	0.000	0.001	0.002	0.000	0.000
	BS00104177_51	3B	CT	TT	128	34	8.41	9.59	0.000	0.000	0.000	0.000	0.000	0.000	0.000	0.000
	Kukri_rep_c110544_52	3B	TT	CT	128	34	8.40	9.59	0.000	0.000	0.000	0.000	0.000	0.000	0.000	0.000
	Excalibur_c20309_539	3B	TT	CT	128	34	8.40	9.59	0.000	0.000	0.000	0.000	0.000	0.000	0.000	0.000
	BS00000020_51	5DS	CC	TT	71	81	7.83	9.42	0.000	0.000	0.000	0.000	0.000	0.000	0.000	0.000
	Kukri_rep_c68371_1242	7AL	AG	GG	88	72	8.14	9.28	0.000	0.000	0.000	0.000	0.000	0.000	0.000	0.000
	tplb0026f23_1134	7BL	AA	GG	110	23	8.40	10.52	0.000	0.000	0.000	0.000	0.000	0.000	0.000	0.000
	Kukri_c4352_194	7BL	AA	GA	141	22	8.35	10.55	0.000	0.000	0.000	0.000	0.000	0.000	0.000	0.000
	RAC875_c38693_499	7BL	TT	CT	139	22	8.34	10.55	0.000	0.000	0.000	0.000	0.000	0.000	0.000	0.000
	Excalibur_c5851_1661	7BL	TT	CT	134	17	8.28	10.55	0.000	0.000	0.000	0.000	0.000	0.000	0.000	0.000
	Excalibur_rep_c92684_578	7DL	AA	GA	136	22	8.32	10.55	0.000	0.000	0.000	0.000	0.000	0.000	0.000	0.000

Fa*, flour redness; Fb*, flour yellowness; FL*, flour lightness; GPC, grain protein content; HI, hardness index; WGC, wet gluten content.

E1, 2013 Anyang; E2, 2013 Zhengzhou; E3, 2013 Zhumadian; E4, 2014 Anyang; E5, 2014 Zhengzhou; E6, 2014 Zhumadian; E7, 2015 Zhengzhou; E8, 2016 Zhengzhou.

Among the multienvironment‐significant SNPs, some of them were significantly associated with more than one trait. We made a validation for pleiotropic SNPs associated with two or more traits (Table S8). The *t*‐test indicated that cultivars with allele TT at BS00000020_51 (i.e. *Pinb*) locus showed significantly higher HI, Fb* and lower FL*, whereas cultivars with allele GG at IAAV7827 showed lower HI and higher FL*. Cultivars with allele GG at BS00046964_51, AG at IAAV5188 and CT at RAC875_c3187_873 showed higher GPC and WGC. Cultivars with allele TT at Ex_c16529_304, Kukri_rep_c110544_52, Excalibur_c20309_539, RAC875_c38693_499 and Excalibur_c5851_1661 loci, GA at Excalibur_c42979_666, CT at BS00104177_51 and AA at Kukri_c4352_194, tplb0026f23_1134 and Excalibur_rep_c92684_578 loci showed significantly higher Fa* and lower Fb* (Tables 2, S8).

### QTL mapping

**Figure 4:**
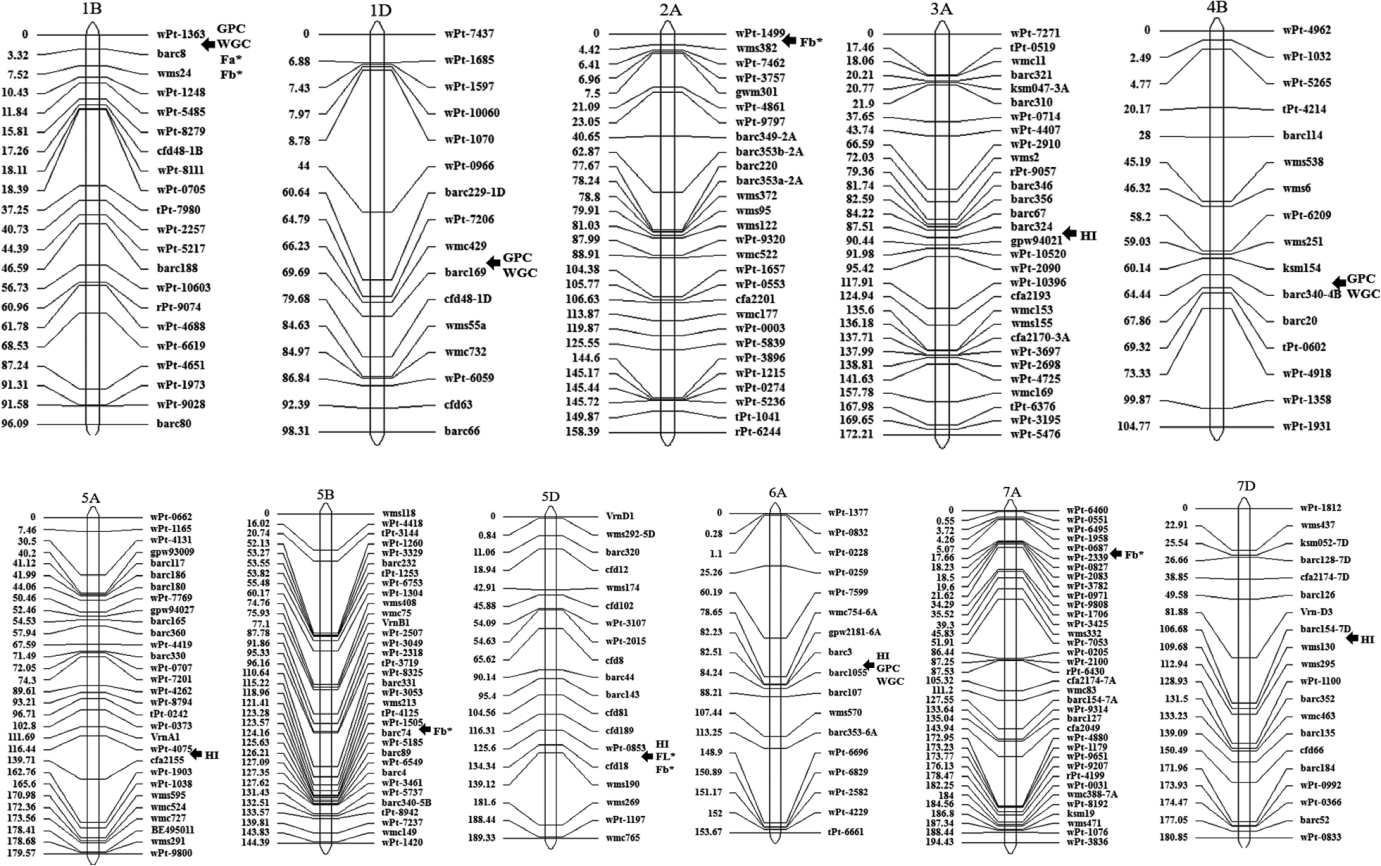
Quantitative trait loci mapping of the six quality traits in a F_10_ RIL population.

Quantitative trait loci mapping for quality traits in the UP population (Table 3, Figure 4) showed that five QTLs for HI were detected on chromosomes 3A, 5A, 5D, 6A and 7D, explaining the PVE of 5.3%–14.1%. Some important SNPs were also significantly associated with HI on 5A and 5D in the GWAS. For GPC and WGC, four QTLs were detected on 1B, 1D, 4B and 6A, with the PVE of 5.1%–12.4%, especially for the QTL located in the marker interval barc3‐barc1055 on 6A with the highest PVE. The GWAS also found many important SNPs on 6AS for GPC and WGC. For flour colour parameters, a major QTL between wPt‐0853 and cfd18 on 5D with PVE of 19.3% was significantly associated with FL*. A QTL between wPt‐0853 and barc8 on 1B was detected for Fa* with PVE 6.3%. For Fb*, five QTLs were mapped on chromosomes 1B, 2A, 5B, 5D and 7A, and explained 5.0–11.0 of the phenotypic variations. Among them, the QTL in the interval wPt‐0853‐cfd18 explained the highest PVE for Fb*.

**Table 3:** QTL mapping for quality traits in the UP population

Trait	Chromosome	Position	Marker interval	Physical location	LOD	PVE	ADD
HI	3A	89	barc324‐gpw94021	≈486122358	8.1	11.5	4.9
	5A	127	wPt‐4075‐cfa2155	≈632596738	2.6	5.7	3.5
	5D	132	wPt‐0853‐cfd18	≈5597656	8.0	12.1	5.1
	6A	84	barc3‐barc1055	191587658‐383858366	4.0	5.3	3.3
	7D	109	barc154‐7D‐wms130	58259360‐62937508	9.7	14.1	−5.5
GPC	1B	3	wPt‐1363‐barc8	≈42329007	3.3	5.6	−0.3
	1D	67	wmc429‐barc169	277305968‐328440737	3.4	5.9	−0.3
	4B	61	ksm154‐barc340‐4B	≈437936771	5.4	9.7	0.4
	6A	84	barc3‐barc1055	191587658‐383858366	5.7	10.3	−0.4
WGC	1B	3	wPt‐1363‐barc8	≈42329007	3.1	5.1	−0.6
	1D	67	wmc429‐barc169	277305968‐328440737	2.9	4.9	−0.6
	4B	62	ksm154‐barc340‐4B	≈437936771	4.9	9.1	0.8
	6A	84	barc3‐barc1055	191587658‐383858366	6.9	12.4	−0.9
FL*	5D	134	wPt‐0853‐cfd18	≈5597656	8.1	19.3	−0.4
Fa*	1B	0	wPt‐1363‐barc8	≈42329007	2.5	6.3	0.0
Fb*	1B	1	wPt‐1363‐barc8	≈42329007	2.9	5.0	−0.2
	2A	4	wPt‐1499‐wms382	≈772967310	3.8	6.5	−0.2
	5B	124	wPt‐1505‐barc74	≈402787119	3.4	5.7	0.2
	5D	136	wPt‐0853‐cfd18	≈5597656	5.8	11.0	0.3
	7A	7	wPt‐0687‐wPt‐2339	–	4.8	9.6	0.2

Fa*, flour redness; Fb*, flour yellowness; FL*, flour lightness; GPC, grain protein content; HI, hardness index; WGC, wet gluten content.

Position (cM), distance between QTL and the top marker of each linkage map. Physical location, the location in the entire genome database of Chinese Spring. LOD, a threshold of 2.5 was fixed for declaring the presence of QTL. PVE (%), phenotypic variation explained. ADD, positive values indicate an increasing effect from UC1110; negative values indicate an increasing effect from PI610750.

Some QTLs are related to multiple traits. On 5D, the QTL between wPt‐0853 and cfd18 was simultaneously significantly associated with HI, FL* and Fb*. Further analysis indicated that the *Pina* and *Pinb* genes were close to the marker cfd18 in the genome of AK 58. On 1B, the QTL between wPt‐1363 and barc8 was simultaneously significantly associated with GPC, WGC, Fa* and Fb*. On 6A, the QTL between barc3 and barc1055 was simultaneously significantly associated with HI, GPC and WGC, suggesting that they are important loci to modulate quality traits in bread wheat.

### Association of the *TaRPP13L1* gene with flour colour parameters

**Figure 5:**
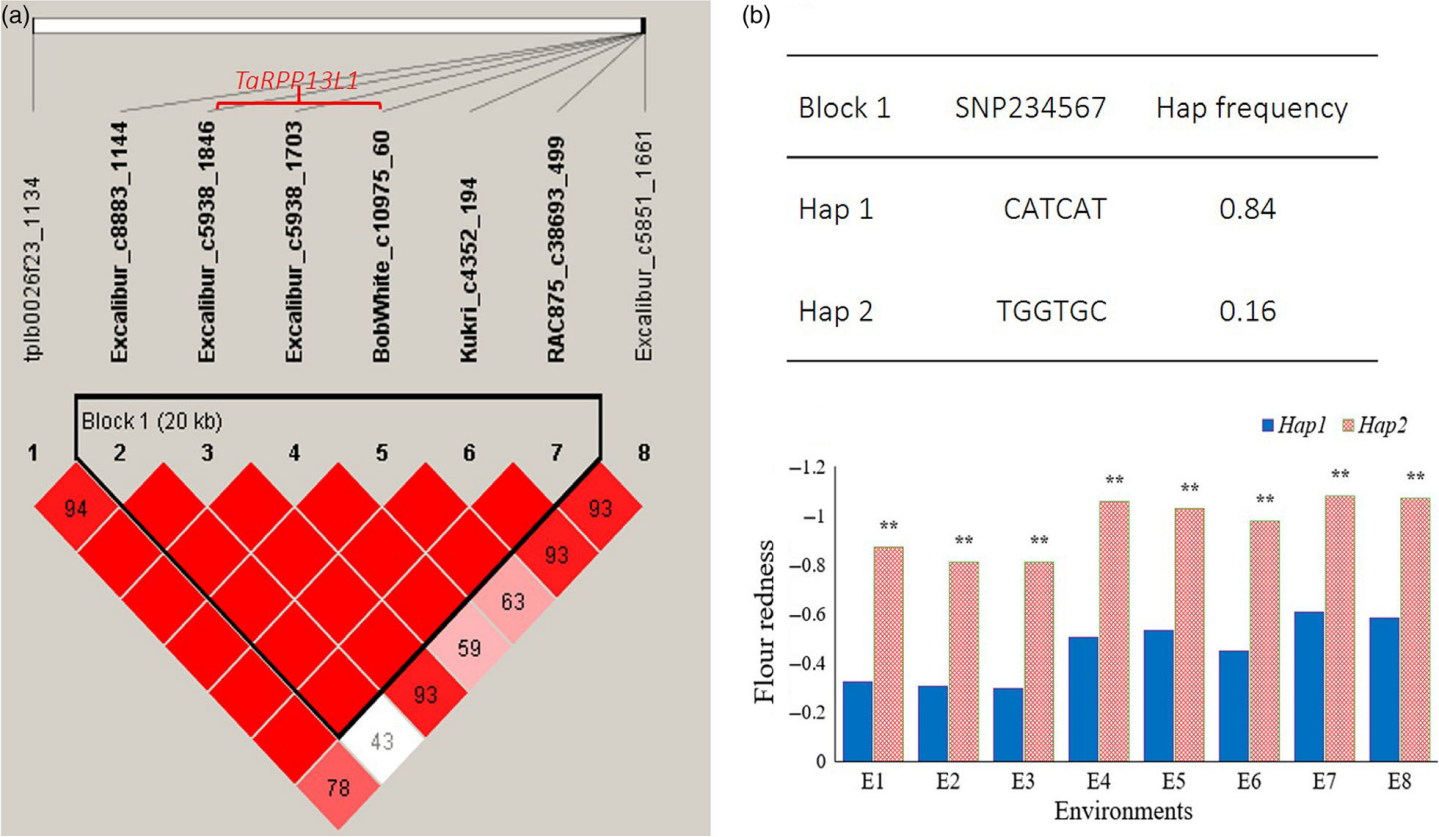
Haplotype analysis of multienvironment‐significant SNPs on 7BL for flour redness.

For Fa*, a haplotype analysis was performed using the 8 significant SNPs on 7BL. The result showed that six SNPs (Excalibur_c8883_1144, Excalibur_c5938_1846, Excalibur_c5938_1703, BobWhite_c10975_60, Kukri_c4352_194 and RAC875_c38693_499) were in a 20‐kb block, and there were two main haplotypes for this block. The haplotype CATCAT possessed extremely significantly higher flour redness than haplotype TGGTGC in all 8 environments (Figure 5).

Further analysis of the sequence in the block indicated that this block contained a complete gene, the disease resistance RPP13‐like protein 1 (*RPP13L1*) gene, which was predicted from *Arabidopsis thaliana*. Excalibur_c5938_1846, Excalibur_c5938_1703 and BobWhite_c10975_60 on 7BL were located in this gene (Figure 5). Kukri_c65663_642 and RAC875_c14064_308 on 7DL were also located on homologous *RPP13L1‐7D* genes in the Chinese Spring and AK58 genome databases (Table 1). On 7BL, three SNPs (Excalibur_c5938_1846, Excalibur_c5938_1703 and BobWhite_c10975_60) were closely linked to the *RPP13L1‐7B* gene. The SNPs in Excalibur_c5938_1846 and BobWhite_c10975_60 did not cause amino acid changes, but Excalibur_c5938_1703 showed T to G changes at the 2905‐bp position of the *TaRPP13L1‐B1* gene, and thus, it resulted in the change in serine to alanine at amino acid position 868. Here, the *TaRPP13L1‐B1* allele with AA in Excalibur_c5938_1846, TG in Excalibur_c5938_1703 and CT in BobWhite_c10975_60 was designated as *TaRPP13L1‐B1a*, and the allele with GA in Excalibur_c5938_1846, GG in Excalibur_c5938_1703 and TT in BobWhite_c10975_60 was designated as *TaRPP13L1‐B1b*. Full alignment of *RPP13L1* genes and their allelic variations in different genomes is shown in Figure S8.

**Figure 6:**
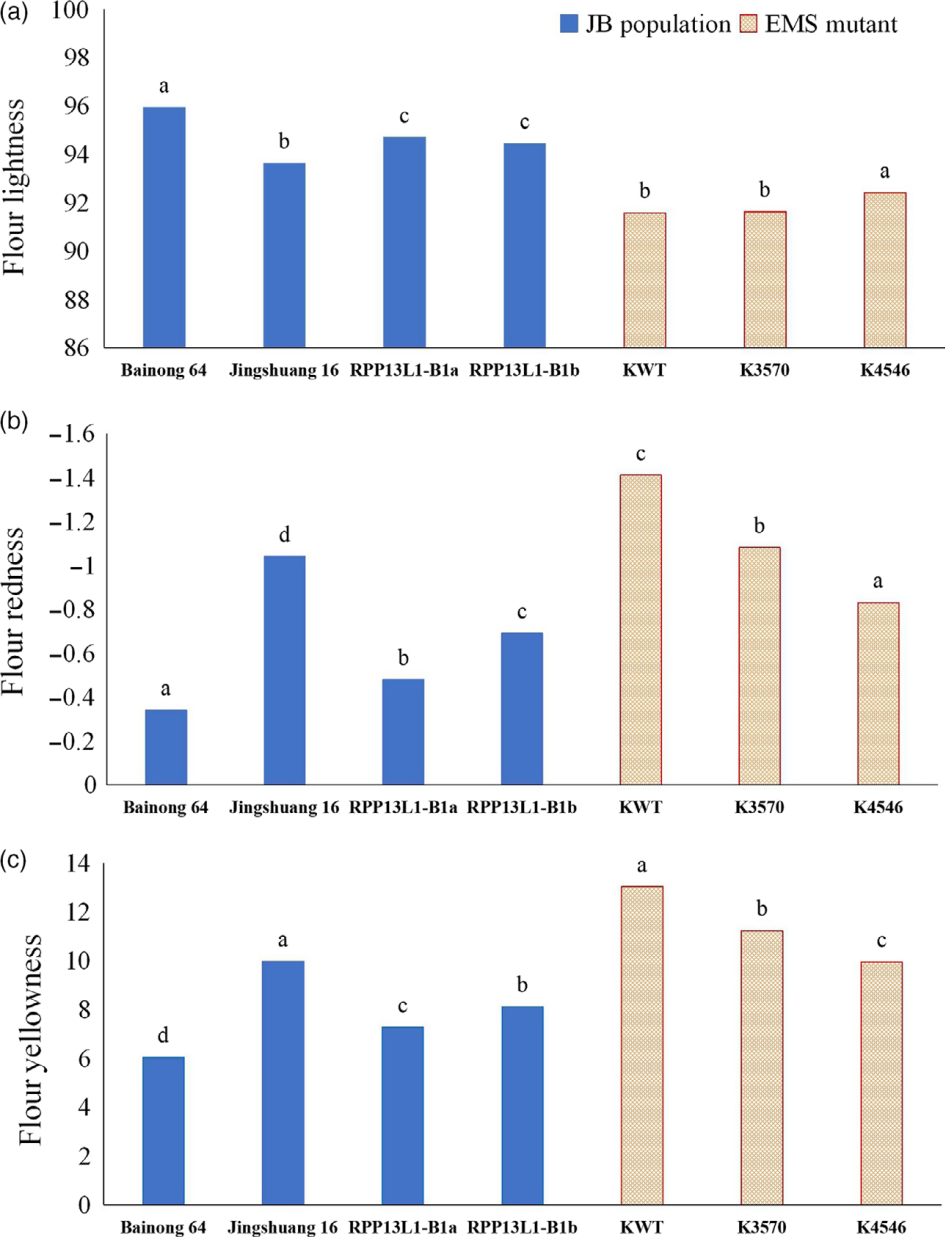
The FL*, Fa* and Fb* in the BJ population and the EMS mutants. Different letters on the column indicate significant differences at the 0.05 level.

In the natural population, wheat cultivars were genotyped with the function marker YP7B‐1 (He *et al*., 2009). The results showed that cultivars with *RPP13L1‐B1a* and *RPP13L1‐B1b* showed significant difference in either *Psy‐B1a* or *Psy‐B1b* genotypes (Table S9). In the BJ population, the parents Bainong 64 and Jingshuang 16 possessed the *RPP13L1‐B1a* and *RPP13L1‐B1b* alleles, respectively, whereas they both had the same *Psy‐B1b* allele. Determination of flour colour parameters indicated that Bainong 64 showed extremely significantly higher flour redness and lower yellowness than Jingshuang 16. In the offspring segregating population, all 95 lines with the *RPP13L1‐B1a* allele also showed averaged significantly higher Fa* and lower Fb* than the 48 lines with the *RPP13L1‐B1b* allele (Table 5, Figure 6).

### EMS mutants

The *TaRPP13L1* gene was further analysed in the repository of Kronos mutants (http://dubcovskylab.ucdavis.edu/). The mutant line Kronos3570 showed a G to A mutation at the 3051‐bp position of the *TaRPP13L1‐A1* gene, and thus, it resulted in a tryptophan stop codon at amino acid position 1016. The mutant line Kronos4546 showed a G to A mutation at the 3039‐bp position of the *TaRPP13L1‐B1* gene, and thus, it resulted in a tryptophan stop codon at amino acid position 1012. These mutations both caused premature stop codons in the *TaRPP13L1* gene. Determination of flour colour parameters of Kronos3570 and Kronos4546 indicated that both mutant lines Kronos4546 and Kronos3570 possessed significantly higher Fa* (−0.83 and −1.08, respectively) and lower Fb* (9.95 and 11.23, respectively) than the wild cultivar (−1.41 for Fa* and 13.03 for Fb*, respectively; Table 5, Figure 6). These data suggest that the *TaRPP13L1* gene significantly regulates flour colour parameters.

### Pyramid effect of multienvironment‐significant SNPs

Multienvironment‐significant SNPs were considered to play key roles in modulating quality traits. We investigated the allele distribution and its pyramid effect of these loci (in more than five environments) in the natural population (Table 4). As shown in Table 4, 21 cultivars containing the 5 alleles with a relatively high HI at the 5 loci (Kukri_c34553_89, wsnp_Ku_c4078_7436510, IAAV7827, Kukri_rep_c102608_599 and BS00000020_51) possessed an average of 67.2 (ranging from 51.3 to 80.0), whereas 7 cultivars containing the alleles with a relatively low HI at the 5 loci exhibited an average of 39.5 (ranging from 26.5 to 65.2).

**Table 4:** Pyramid effect of multienvironment‐significant SNPs for each quality trait

Traits	Alleles	Number of cultivars	Average	Total average
HI†	CC/TC/AA/TC/TT	21	67.2a	54.1
	TC/CC/GG/CC/CC	7	39.5b	
GPC‡	GG/CT/GG/AG/GG/GG/CC/CC/AA/GG/AA	12	14.3%a	13.5%
	AG/TT/AG/GG/AG/AA/TT/AC/GA/AG/GA	3	12.4%b	
WGC§	CT/GG/AG	18	31.0%a	29.7%
	TT/AG/GG	61	28.9%b	
FL*¶	GG/GG/CC/GG/CC	31	96.0a	95.3
	AA/AG/TC/AA/TT	13	94.9b	
Fa*††	TT/GA/CT/TT/TT/CT/AG/AA/CT/AA/TG/CT/AA/TT/TT/GA/TC/AA/TT/AA	32	−0.38a	−0.51
	CC/AA/TT/CT/CT/TT/GG/GG/TT/GA/GG/TT/GA/CT/CT/AA/CC/GA/CT/GA	2	−1.17b	
Fb*[Link]	TT/GA/CT/AC/CT/TT/TT/CC/AG/AA/AA/TT/TT/AA	24	7.3b	8.6
	CC/AA/TT/CC/TT/CT/CT/TT/GG/GG/GA/CT/CT/GA	1	11.8a	

† Kukri_c34553_89, wsnp_Ku_c4078_7436510, IAAV7827, Kukri_rep_c102608_599 and BS00000020_51.

‡ RFL_Contig2972_2819, RAC875_c3187_873, BS00046964_51, IAAV5188, Ra_c28284_223, Tdurum_contig51717_1582, BS00010811_51, wsnp_Ex_c24958_34212226, wsnp_Ex_c43412_49738738, BS00010441_51 and BS00043554_51.

§ RAC875_c3187_873, BS00046964_51 and IAAV5188.

¶ BS00029347_51, BS00041219_51, wsnp_Ra_c24707_34262900, IAAV7827 and BS00000020_51.

†† Ex_c16529_304, Excalibur_c42979_666, BS00104177_51, Kukri_rep_c110544_52, Excalibur_c20309_539, BS00003703_51, RFL_Contig2982_586, tplb0026f23_1134, Excalibur_c8883_1144, Excalibur_c5938_1846, Excalibur_c5938_1703, BobWhite_c10975_60, Kukri_c4352_194, RAC875_c38693_499, Excalibur_c5851_1661, Kukri_c65663_642, RAC875_c14064_308, Excalibur_c8883_214, RAC875_c61016_73 and Excalibur_rep_c92684_578.

‡‡ Ex_c16529_304, Excalibur_c42979_666, RAC875_rep_c117294_342, RAC875_rep_c118396_333, BS00104177_51, Kukri_rep_c110544_52, Excalibur_c20309_539, BS00000020_51, Kukri_rep_c68371_1242, tplb0026f23_1134, Kukri_c4352_194, RAC875_c38693_499, Excalibur_c5851_1661 and Excalibur_rep_c92684_578.

Different letters after numbers indicate significant differences at the 0.05 level.

For GPC, 12 cultivars containing the alleles with a relatively high GPC at the 11 loci (RFL_Contig2972_2819, RAC875_c3187_873, BS00046964_51, IAAV5188, Ra_c28284_223, Tdurum_contig51717_1582, BS00010811_51, wsnp_Ex_c24958_34212226, wsnp_Ex_c43412_49738738, BS00010441_51 and BS00043554_51) showed an average of 14.3% (ranging from 13.3% to 15.8%), whereas 3 cultivars without any alleles showing relatively high GPC exhibited an average of 12.4% (ranging from 12.0% to 13.0%).

For WGC, 18 cultivars containing the alleles with a relatively high WGC at the three loci (RAC875_c3187_873, BS00046964_51 and IAAV5188) showed an average of 31.0% (ranging from 28.4% to 34.1%), whereas 61 cultivars containing the alleles with a relatively low WGC exhibited an average of 28.9% (ranging from 25.9% to 32.4%).

For FL*, 31 cultivars containing all alleles with relatively high flour lightness at five loci (BS00029347_51, BS00041219_51, wsnp_Ra_c24707_34262900, IAAV7827 and BS00000020_51) showed an average of 96.0 (ranging from 94.2 to 96.6), whereas 13 cultivars containing all alleles showing relatively low flour lightness exhibited an average of 94.9 (ranging from 94.3 to 95.6).

For Fa*, 32 cultivars containing all alleles with relatively high flour redness at the 20 loci (Ex_c16529_304, Excalibur_c42979_666, BS00104177_51, Kukri_rep_c110544_52, Excalibur_c20309_539, BS00003703_51, RFL_Contig2982_586, tplb0026f23_1134, Excalibur_c8883_1144, Excalibur_c5938_1846, Excalibur_c5938_1703, BobWhite_c10975_60, Kukri_c4352_194, RAC875_c38693_499, Excalibur_c5851_1661, Kukri_c65663_642, RAC875_c14064_308, Excalibur_c8883_214, RAC875_c61016_73 and Excalibur_rep_c92684_578) showed an average of −0.38 (ranging from −0.86 to −0.01), whereas the 2 cultivars containing all alleles with relatively low flour redness exhibited −1.17.

For Fb*, cultivar Jingshuang 16 with all alleles showing relatively high flour yellowness at 14 loci (Ex_c16529_304, Excalibur_c42979_666, RAC875_rep_c117294_342, RAC875_rep_c118396_333, BS00104177_51, Kukri_rep_c110544_52, Excalibur_c20309_539, BS00000020_51, Kukri_rep_c68371_1242, tplb0026f23_1134, Kukri_c4352_194, RAC875_c38693_499, Excalibur_c5851_1661 and Excalibur_rep_c92684_578) exhibited 11.8, whereas 24 cultivars containing all alleles with relatively low flour yellowness showed an average of 7.3 (ranging from 5.9 cm to 9.1). These data suggest that pyramiding multiple above‐mentioned SNPs loci for each quality trait is more effective for improvement of wheat quality in Chinese wheat breeding programmes.

### Development of dCAPS markers

**Figure 7:**
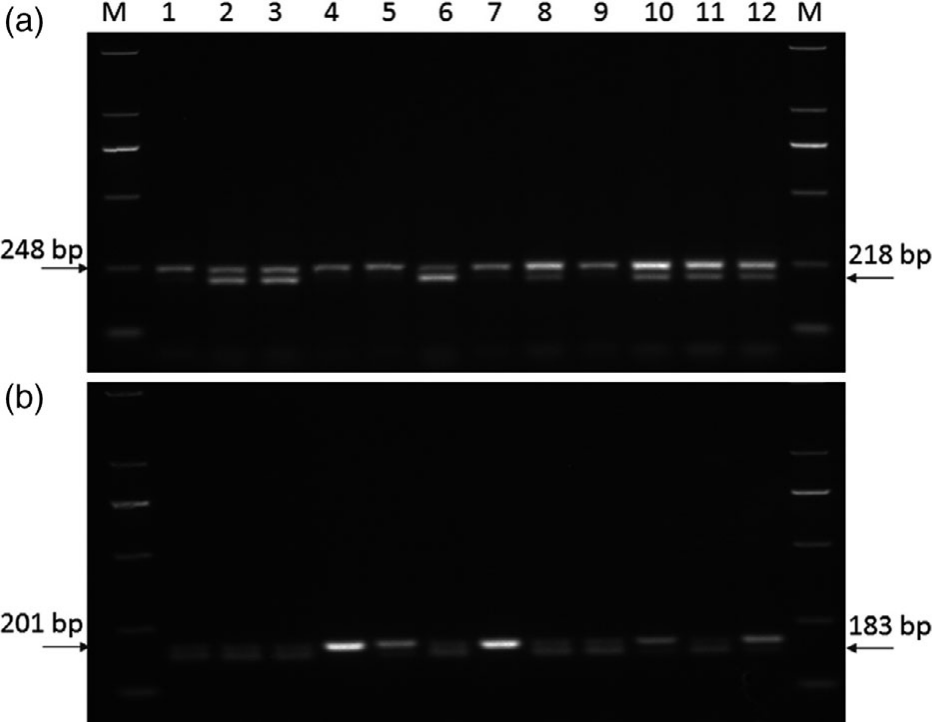
Development of molecular marker to distinguish genotypes for quality traits. (a) The dCAPS marker for identification of alleles at the BS00046964_51 locus, (b) the dCAPS marker for identification of *TaRPP13L1‐B1a* and *TaRPP13L1‐B1b* alleles. M: DNA Ladder DL2000.

Based on sequences of the *TaRPP13L1‐B1a* and *TaRPP13L1‐B1b* alleles, a dCAPS marker was developed, and cultivars with an 183‐bp fragment possessed *TaRPP13L1‐B1a* allele by the digestion of restriction enzyme *SphI* (Figure 7b, Table S10). Cultivars or lines with *TaRPP13L1‐B1a* showed significantly higher Fa* than those with *TaRPP13L1‐B1b* (Tables 2, 5).

**Table 5:** The FL*, Fa* and Fb* in the BJ population and the EMS mutants

Allele	Number	FL*	Fa*	Fb*
Bainong 64	–	95.94a	−0.34a	6.04d
Jingshuang 16	–	93.63c	−1.04d	9.97a
*RPP13L1‐B1a*	95	94.70b	−0.48b	7.28c
*RPP13L1‐B1b*	48	94.45b	−0.69c	8.11b
KWT	–	91.58b	−1.41c	13.03a
K3570	–	91.63b	−1.08b	11.23b
K4546	–	92.42a	−0.83a	9.95c

Different letters after numbers indicate significant differences at the 0.05 level.

BS00046964_51 on 6AS was the most significant for GPC and WGC in multiple environments, and one dCAPS marker was developed for identification of two alleles at the BS00046964_51 locus. Cultivars with BS00046964_51_*GG* produced a 218‐bp digestion fragment by restriction enzyme *NaeI*, and cultivars with BS00046964_51_*AG* were not digested by *NaeI* (Figure 7b, Table S10). Cultivars with BS00046964_51_*GG* possessed higher GPC (13.8) and WGC (30.3) than cultivars with BS00046964_51_*AG* (13.2 for GPC and 29.1 for WGC; Table 2).

BS00000020_51 on 5DS was the most significant in all 8 environments for HI, FL* and Fb*. Further analysis indicated that BS00000020_51 was part of *Pinb‐D1* gene, and its marker has been developed in previous study (Giroux and Morris, 1997). Cultivars with BS00000020_51_*TT* (*Pinb‐D1b*) showed significantly higher HI (64.5) and Fb* (9.42), and lower FL* (94.9) than cultivars with BS00000020_51_*CC* (*Pinb‐D1a*; 42.1 for HI, 7.83 for Fb* and 95.7 for FL*; Tables 2, S10).

## Discussion

As the most important and largest wheat production zone in China, the Yellow and Huai River wheat region also possesses unique ecological conditions for producing high‐quality special wheat. At present, improving quality and expanding the planting area of high‐quality wheat have become increasingly important. Henan wheat production accounts for approximately a quarter of the total production in China. According to the ‘Henan Province's Work Plan on Promoting the Development of High‐quality Wheat (2017–2018)’, most areas focus on producing high‐gluten, high‐quality wheat, and some areas along the Huai River south of Henan focus on planting soft low‐gluten, high‐quality wheat. Therefore, revealing the genetic basis of quality traits and improving quality are of great significance in this wheat region.

Previous studies indicated that the *Pina* and *Pinb* genes on chromosome 5DS in the *Ha* locus mainly control grain hardness in bread wheat (Chen *et al*., 2013; Morris *et al*., 2001). In the current study, the SNP BS00000020_51 on chromosome 5DS is part of the *Pinb* gene, which was most significant and detected in all of the 8 environments, and explained the 40.1% phenotypic variation. The allele CC at this locus is from the wild‐type gene *Pinb‐D1a*, and the allele TT is from the gene *Pinb‐D1b*. Additionally, the hardness index (HI) of *Pinb‐D1b* is significantly higher than that of *Pinb‐D1a*, which is consistent with the results of previous studies (Chen *et al*., 2007; Giroux and Morris, 1997). IAAV7827 and Kukri_rep_c102608_599 on chromosome 5AL were significant in 8 and 7 environments, respectively. In the RIL population UP, one QTL explaining 5.7% phenotypic variation was also detected on 5A (Table 3). Sourdille *et al*. (1996), Li *et al*. (2009) and Bordes *et al*. (2011) also detected some QTLs on 5A in the previous studies, which indicates that there is at least one important genetic locus on chromosome 5AL to modulate grain hardness. We also detected a SNP (wsnp_Ku_c4078_7436510) on 3B to be significantly associated with grain hardness, which was consistent with Bordes *et al*. (2011) and Li *et al*. (2016).

Grain protein content and wet gluten content (WGC) are important quality indicators for wheat. In the current study, 7 multienvironment‐significant SNPs on 6AS were significantly associated with GPC, and 2 of them were also significantly associated with WGC. Previous studies and our QTL mapping also detected some QTLs on 6A for GPC (Bordes *et al*., 2011; Li *et al*., 2012; Perretant *et al*., 2000) and WGC (Plessis *et al*., 2013). Therefore, there is at least one important genetic locus on 6AS that plays an important role in regulating GPC and WGC. On 2DL, RAC875_c3187_873 was significantly associated with GPC and WGC in multiple environments. There may be a genetic locus on 2DL to control GPC and WGC.

Flour colour is considered an important quality trait in determining the end use of wheat. In this study, SNPs significantly associated with FL* were mainly observed on chromosomes 2DS, 3B, 5AS, 5AL, 5DS and 5DL. Among them, the SNP BS00000020_51 (i.e. *Pinb*) on 5DS was extremely significantly detected in all 8 of the environments. The FL* of *Pinb‐D1a* is significantly higher than the FL* of *Pinb‐D1b*, which is consistent with a previous study (Chen *et al*., 2007). In addition, three SNPs (BS00029347_51, BS00041219_51 and wsnp_Ra_c24707_34262900) on 5AS were all detected in 7 environments, suggesting that there is at least one genetic locus on 5AS that is associated with FL*. On 5AL, IAAV7827 was significantly associated with flour lightness and hardness, indicating that there is an intimate relationship between flour lightness and hardness. Some QTLs were also mapped on 5A in a previous study (Tsilo *et al*., 2011) and the current study, and therefore, there are some FL*‐related important genetic loci on 5A.

SNPs significantly associated with Fb* were mainly observed on chromosomes 1AL, 2AS, 2AL, 2BL, 3B, 4AL, 5BL, 5DS, 6BS, 7AL, 7BL and 7DL. On 5DS, the SNP BS00000020_51 (i.e. *Pinb*) was also detected in all 8 environments and was significantly associated with HI and FL*, which is consistent with a previous study (Chen *et al*., 2007). These data show that the *Pinb* gene simultaneously regulates HI, FL* and Fb*. The results of the association analysis also showed that the three traits were extremely related to each other (Table S1). On 7BL, tplb0026f23_1134 (i.e. *Psy*) was also significantly associated with flour yellowness. In addition, Excalibur_c42979_666 (it may be linked to *PPO* gene) on 2AL was detected in all 8 environments and extremely significantly associated with Fa* and Fb*.

Zhang and Dubcovsky (2008b) reported that *Psy‐E1*, a tall wheatgrass orthologue, was completely linked to grain yellow pigment content (GYPC) and was further used to select for white endosperm mutants in recombinant lines carrying a mutant of the *Psy‐E1* gene. The results showed an association of GYPC with allelic variations of *Psy‐1* gene in hexaploidy wheat. However, a white endosperm mutant mapped to chromosome arm 7EL showed no mutations in *Psy‐E1*, suggesting the existence of additional gene(s) affecting GYPC in 7EL. This hypothesis was further supported by the mapping of QTL for GYPC on 7AL proximal to *Psy‐1* in a cross of durum wheat UC1113 and Kofa. Therefore, the authors speculated that *Psy‐1* and at least one additional gene in the distal region of the long arm of homologous group 7 were associated with flour colour. Roncallo *et al*. (2012) also detected one additional QTL on 7BL to be significantly associated with flour colour except for the *Psy‐B1* gene, and the new QTL had a higher PVE than the *Psy‐B1* gene. Actually, it is quite normal to have more than one gene in a small region in bread wheat, for example three genes of *Pina*,* Pinb* and *GSP‐1* related to wheat kernel hardness are identified within the 39‐kb region on 5DS (*Pina* is only 17.6 kb away from *Pinb*). In summary, we concluded that there were at least two important genes affecting flour colour on the 7 homologous chromosomes.

In this study, SNPs significantly associated with Fa* were mainly observed on chromosomes 1AL, 1DL, 2AL, 2DL, 3B, 4AL, 4DS, 7AL, 7BL and 7DL. BS00003703_51 on 7A and tplb0026f23_1134 on 7BL were the *Psy* genes, which had been verified to sharply affect flour colour (Zhang *et al*., 2008a). However, Excalibur_c5938_1846, BobWhite_c10975_60 and Excalibur_c5938_1703 on 7BL, and Kukri_c65663_642 and RAC875_c14064_308 on 7DL, which were homologous *TaRPP13L1* genes, were also significantly associated with Fa*. Therefore, we further analysed the association of the *TaRPP13L1* gene with flour colour parameters in biparent populations and EMS mutants.

Analysis of the physical location indicated that *Psy‐7B* and *TaRPP13L1‐B1* possessed 0.6 Mb distance in the Chinese Spring database and 0.9 Mb distance in the Aikang 58 database. Therefore, it is difficult to separate these two genes in traditional QTL mapping. Cultivars and lines with *TaRPP13L1‐B1a* in the natural and DH populations had extremely significantly higher flour redness and lower yellowness than cultivars and lines with *TaRPP13L1‐B1b*, respectively. Furthermore, two EMS mutant lines with a premature stop codon of the *TaRPP13L1‐B1* gene showed extremely significantly higher flour redness and lower yellowness than wild cultivar. Therefore, combining previous reports and this study, we determined that *TaRPP13L1* on chromosome 7 was a new gene that modulates flour colour in bread wheat.

Phenotypic data collection for quality traits, such as hardness and flour colour in this study, is costly, laborious, time‐consuming and destructive, and thus, breeders generally do not use these methods for selection in early generations (Jernigan *et al*., 2018). Wheat quality traits are predominately controlled by genetic factors (Carter *et al*., 2012), and therefore, it is advantageous to use molecular markers to select experimental lines with superior traits or predict quality indicators. In this study, some novel important genetic loci (e.g. BS00000020_51 for HI, FL* and Fb*; BS00046964_51 for GPC; Excalibur_c42979_666, BS00003703_51, RFL_Contig2982_586, tplb0026f23_1134 and Excalibur_c8883_1144 for Fa*) could be considered for molecular marker‐assisted selection to improve breeding efficiency in the Yellow and Huai River wheat region. In this study, two dCAPS markers were developed and could be used for marker‐assisted selection in wheat quality trait improvement.

The pyramid effect of important genetic loci was effective for rapid improvement of quality traits in this wheat breeding programme. In the current study, the pyramid effect for each of the six traits is very significant. For example, flour with a relatively high b* value is desirable for both Japanese and Chinese yellow noodles (Kruger *et al*., 1992). Cultivar Jingshuang 16 contained all of the alleles showing relatively high flour yellowness at the 14 loci (Ex_c16529_304, Excalibur_c42979_666, RAC875_rep_c117294_342, RAC875_rep_c118396_333, BS00104177_51, Kukri_rep_c110544_52, Excalibur_c20309_539, BS00000020_51, Kukri_rep_c68371_1242, tplb0026f23_1134, Kukri_c4352_194, RAC875_c38693_499, Excalibur_c5851_1661 and Excalibur_rep_c92684_578) and showed Fb* of 11.82, while the averaged flour yellowness of all cultivars surveyed was 8.65. Therefore, these loci could be considered to improve cultivars for alkaline yellow noodles. In short, we can improve the cultivars by pyramiding target gene loci according to our purpose.

## Materials and methods

### Plant materials

Based on pedigree, released regions, agronomic performance, importance (backbone parents or not) and cultivated areas, a total of 163 bread wheat cultivars from the Yellow and Huai Valley of China, including Henan, Hebei, Shaanxi, Shanxi and Shandong Provinces, were selected for the GWAS (Chen *et al*., 2013; Wang *et al*., 2015). These cultivars were planted at the Zhengzhou Scientific Research and Education Center of Henan Agricultural University (34.87°N, 113.5975°E) during the 2012–2013, 2013–2014, 2014–2015 and 2015–2016 cropping seasons, and the Anyang Academy of Agricultural Science (36.10°N, 114.41°E) and the Zhumadian Academy of Agricultural Science (33.01°N, 114.05°E) during the 2012–2013 and 2013–2014 cropping seasons, as we previously described (Sun *et al*., 2017). The field experiment was performed using a randomized complete block design. Each plot contained four 200‐cm‐long rows with 23 cm between neighbouring rows and 10 cm between neighbouring plants. All of the cultivars surveyed were planted at two land parcels in each location. No lodging occurred in field.

A biparental population with 187 F_10_ recombinant inbred lines (RIL) derived from the UC1110/PI610750 (UP) cross was planted at Zhengzhou during the 2015–2016 cropping season. A doubled haploid (DH) population of Bainong 64/Jingshuang 16 (BJ) comprising 143 lines was planted at the Yuanyang Scientific Research and Education Center of Henan Agricultural University (35.11°N, 113.9474°E) during the 2017–2018 cropping season. Each population was planted in a randomized complete block design with two replications. The two populations grew well, and no lodging occurred in field.

### Phenotypic data collection

In order to obtain a sufficient quantity of seeds for accurate assessment of wheat quality, kernels of two replicates for each cultivar were fully mixed together. The kernel hardness index (HI) was measured by the Perten Single Kernel Characterization System (SKCS) 4100 using 300 randomly selected seeds. The grain protein content (GPC) and wet gluten content (WGC) were tested by near‐infrared spectroscopy (NIRS) using a Perten DA 7200 instrument (Perten Instruments, Huddinge, Sweden) under 12% moisture content. Each sample was measured twice, and the average was used for further analysis.

Wheat grains of all of the cultivars surveyed were milled into flour using a Chopin CD1 laboratory mill (Chopin Technologies, Paris, France) according to the method of Chen *et al*. (2013). Flour colour parameters, including flour L* (FL*), flour a* (Fa*) and flour b* (Fb*) for all wheat cultivars surveyed, were measured using a Colorimeter CR 410 (Konica Minolta Holdings, Inc.). The L* value indicates the flour lightness, with a range of 0–100 representing darkness to lightness. The a* value indicates the degree of the flour red–green colour, and the higher a* value denotes a greater amount of red. The b* value indicates the degree of the flour yellow–blue colour, and the higher b* value denotes a greater amount of yellow (Hutchings, 1994). The colorimeter parameter of each sample was measured three times, and the mean values were used for subsequent statistical analyses.

### Genome‐wide association study

All of the surveyed cultivars were genotyped by the Beijing Compass Technology & Investment Co. Ltd. (http://www.bjcompass.com/), using the wheat 90K genotyping assay (Wang *et al*., 2014). Only SNPs with polymorphisms in the association panel were retained for GWAS analysis. Missing genotypes were input and filled using a k‐nearest neighbour‐based imputation algorithm (Huang *et al*., 2010).

The GWASs were implemented with the GAPIT packages (Lipka *et al*., 2012) in R, using the mixed linear model (PCA + K; Yu *et al*., 2006; Zhang *et al*., 2010). The first three principal components were included in the GWAS model. In order to combine the GWAS results in all eight environments, a uniform suggestive genome‐wide significance threshold (*P* value = 1.0e‐3) was given. After the GWAS, significant SNPs with minor allele frequency (MAF) > 0.05 were used for further analysis.

### QTL mapping and haplotype analysis

A genetic linkage map of the UP population was constructed using 558 unique marker loci extracted from 1494 polymorphic probes (SSRs, DArTs and ESTs; Lowe *et al*., 2011) using IciMapping 4.0 software (http://www.isbreeding.net/). Inclusive composite interval mapping of the additive genetic model (ICIM‐ADD) was selected as the mapping model, and other parameters referred to the default design. The threshold for declaring the presence of QTL was fixed at a limit of detection (LOD) value of 2.5. Haplotype analysis was performed using software Haploview 4.2.

### Search for candidate genes with quality‐related traits

The flanking sequences of significant SNPs were blasted in the National Center for Biotechnology Information (NCBI) database (http://www.ncbi.nlm.nih.gov/), the entire genome database of Chinese Spring (http://plants.ensembl.org/Triticum_aestivum/Info/Index) and Aikang 58 (unpublished data) to identify putative genes. The most significant SNP was used to identify candidate genes in its flanking 2 Mb range in the genomes of Chinese Spring and Aikang 58. The dCAPS markers were designed by dCAPS Finder 2.0 (http://helix.wustl.edu/dcaps/dcaps.html).

### EMS mutants of the *TaRPP13L1* gene

Seeds of the tetraploid wheat cultivar Kronos were mutagenized by the group of Jorge Dubcovsky from UC Davis using the chemical mutagen ethyl methanesulfonate (EMS). All of the EMS mutant lines of Kronos were sequenced using exome capture and next‐generation sequencing (Henry *et al*., 2014). The mutant lines Kronos3570 and Kronos4546, kindly provided by Prof. Jorge Dubcovsky, were planted at the Yuanyang Scientific Research and Education Center of Henan Agricultural University during the 2017–2018 cropping season.

## Conflict of interest

The authors declare no conflict of interest.

## Author contributions

F.C. designed the project. J.C., F.Z., G.L. and Y.P. measured quality traits. J.C. and F.C. wrote the manuscript. J.C., C.S., C.Z., X.Y. and F.C. performed the SNP sequencing experiments, QTL mapping and computational analyses.

## Supporting information


**Figure S1** Phenotype distribution of the surveyed cultivars in 8 environments.

**Figure S2** Manhattan and Q–Q plots for HI in 8 environments.

**Figure S3** Manhattan and Q–Q plots for grain protein content in 8 environments.

**Figure S4** Manhattan and Q–Q plots for wet gluten content in 8 environments.

**Figure S5** Manhattan and Q–Q plots for flour L* in 8 environments.

**Figure S6** Manhattan and Q–Q plots for flour a* in 8 environments.

**Figure S7** Manhattan and Q–Q plots for flour b* in 8 environments.

**Figure S8** Full alignment of *TaRPP13L1* genes and their alleles in different genomes.

**Table S1** Pearson's coefficients of correlation based on averaged (A) and each (B) values among quality traits

**Table S2** GWAS result for hardness index in 8 environments

**Table S3** GWAS result for grain protein contents in 8 environments

**Table S4** GWAS result for wet gluten content in 8 environments

**Table S5** GWAS result for flour lightness in 8 environments

**Table S6** GWAS result for flour redness in 8 environments

**Table S7** GWAS result for flour yellowness in 8 environments

**Table S8** T‐test analysis for multienvironment‐significant SNP in different traits

**Table S9** Comparison of flour redness for *TaRPP13L1* and *Psy* alleles in natural population

**Table S10** Development of molecular markers for six quality traits
