# Supplementary material for: Genome‐wide association study of six quality traits reveals the association of the TaRPP13L1 gene with flour colour in Chinese bread wheat
Source: Plant Biotechnol J. 2019 Apr 21;17(11):2106–22. doi: 10.1111/pbi.13126 (PMC6790371; doi:10.1111/pbi.13126)
Supplement: Supplementary file 1 — Figure S1 Phenotype distribution of the surveyed cultivars in 8 environments. [file PBI-17-2106-s008.pdf]

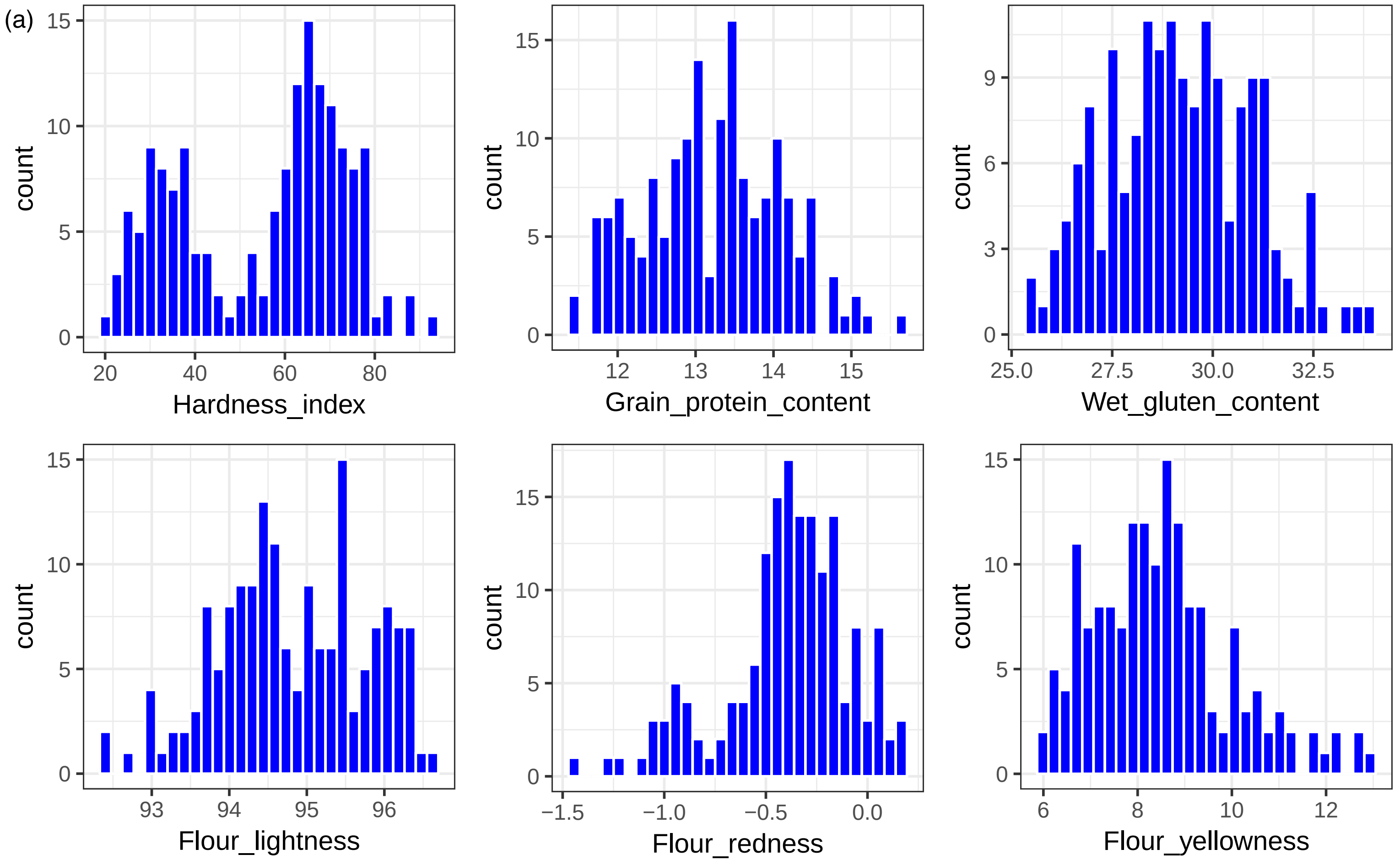
(a)
count
Hardness_index
Grain_protein_content
Wet_gluten_content
Flour_lightness
Flour_redness
Flour_yellowness

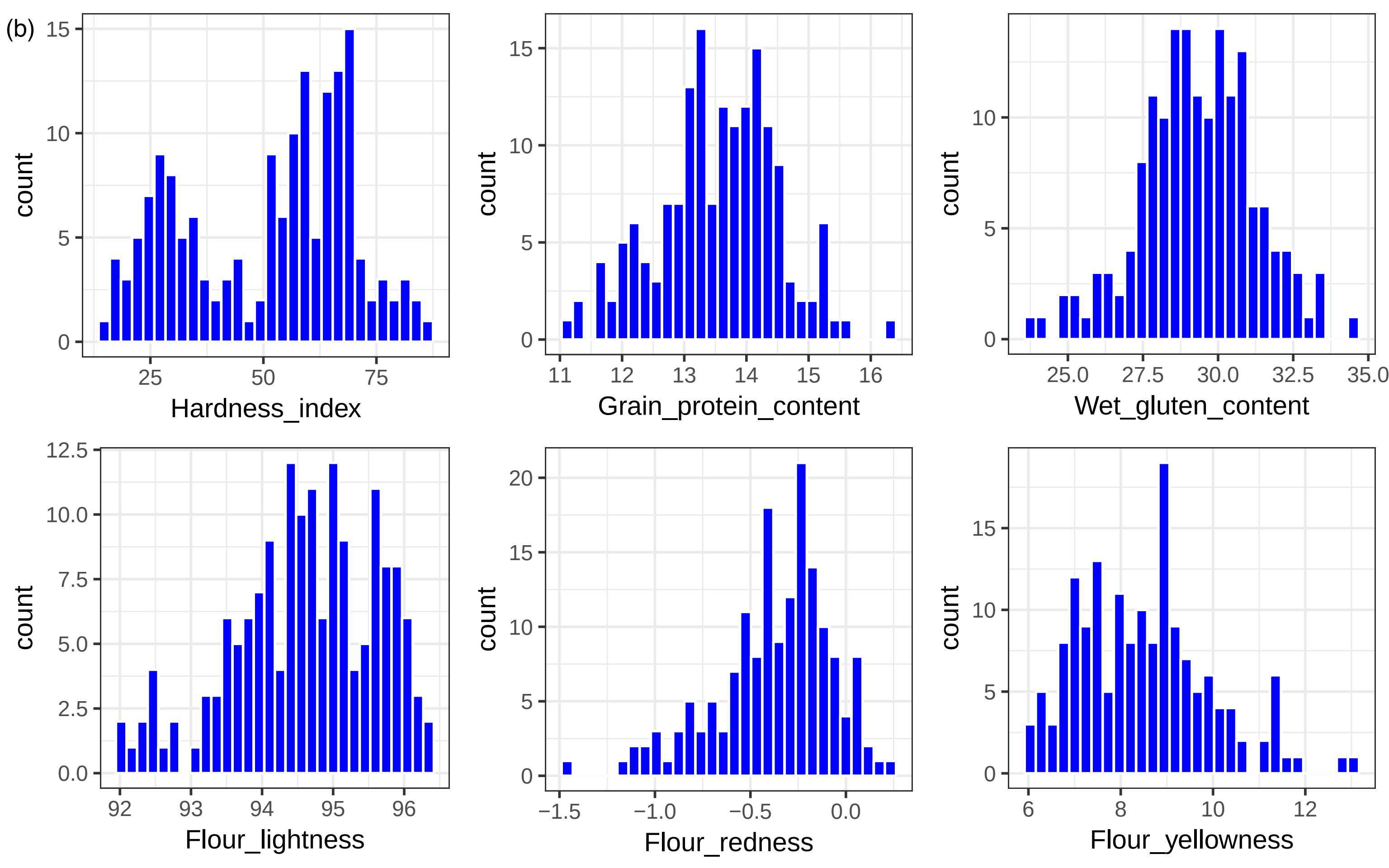
(b)
count
Hardness_index
Grain_protein_content
Wet_gluten_content
Flour_lightness
Flour_redness
Flour_yellowness

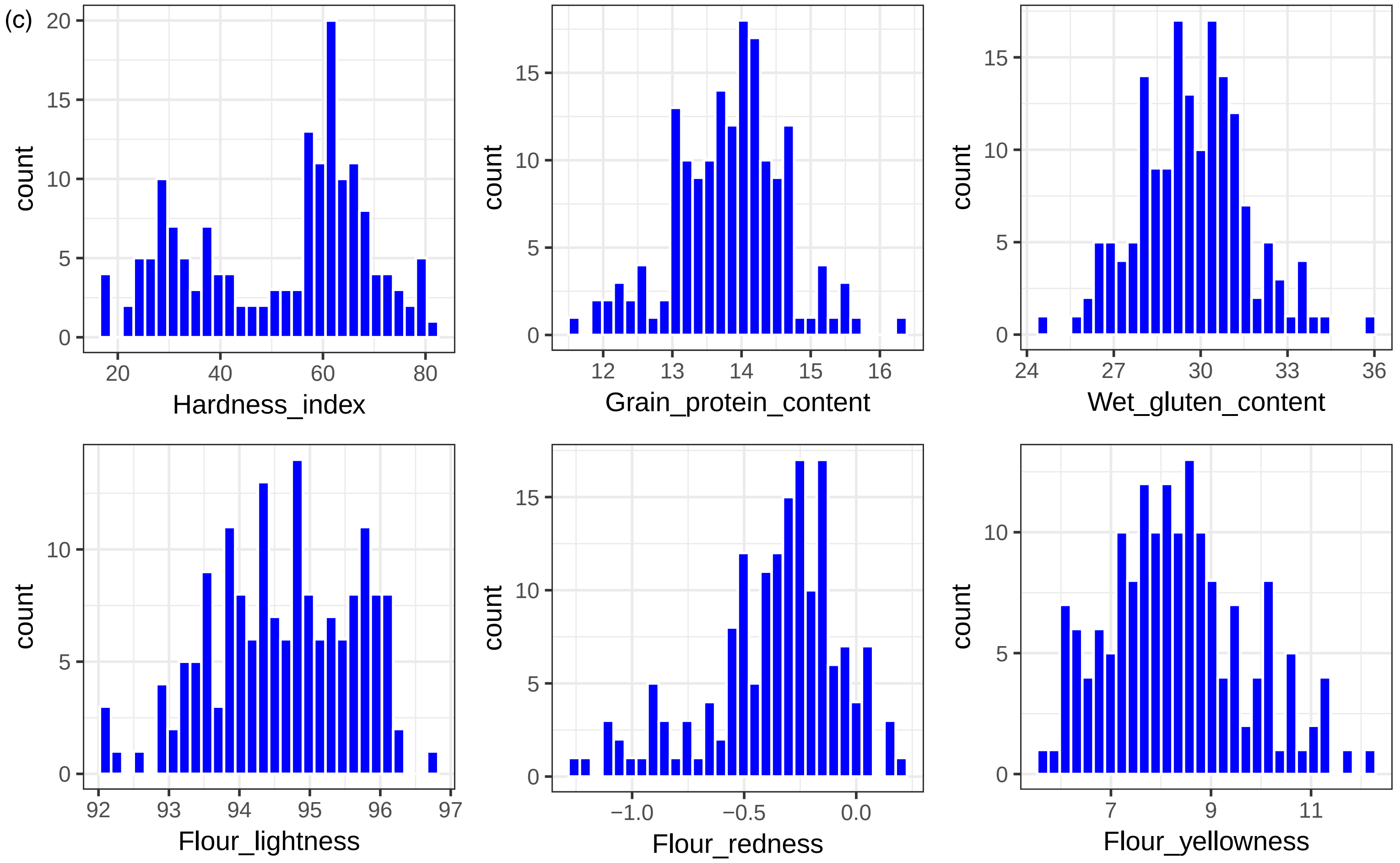
(c)
count
Hardness_index
Grain_protein_content
Wet_gluten_content
Flour_lightness
Flour_redness
Flour_yellowness

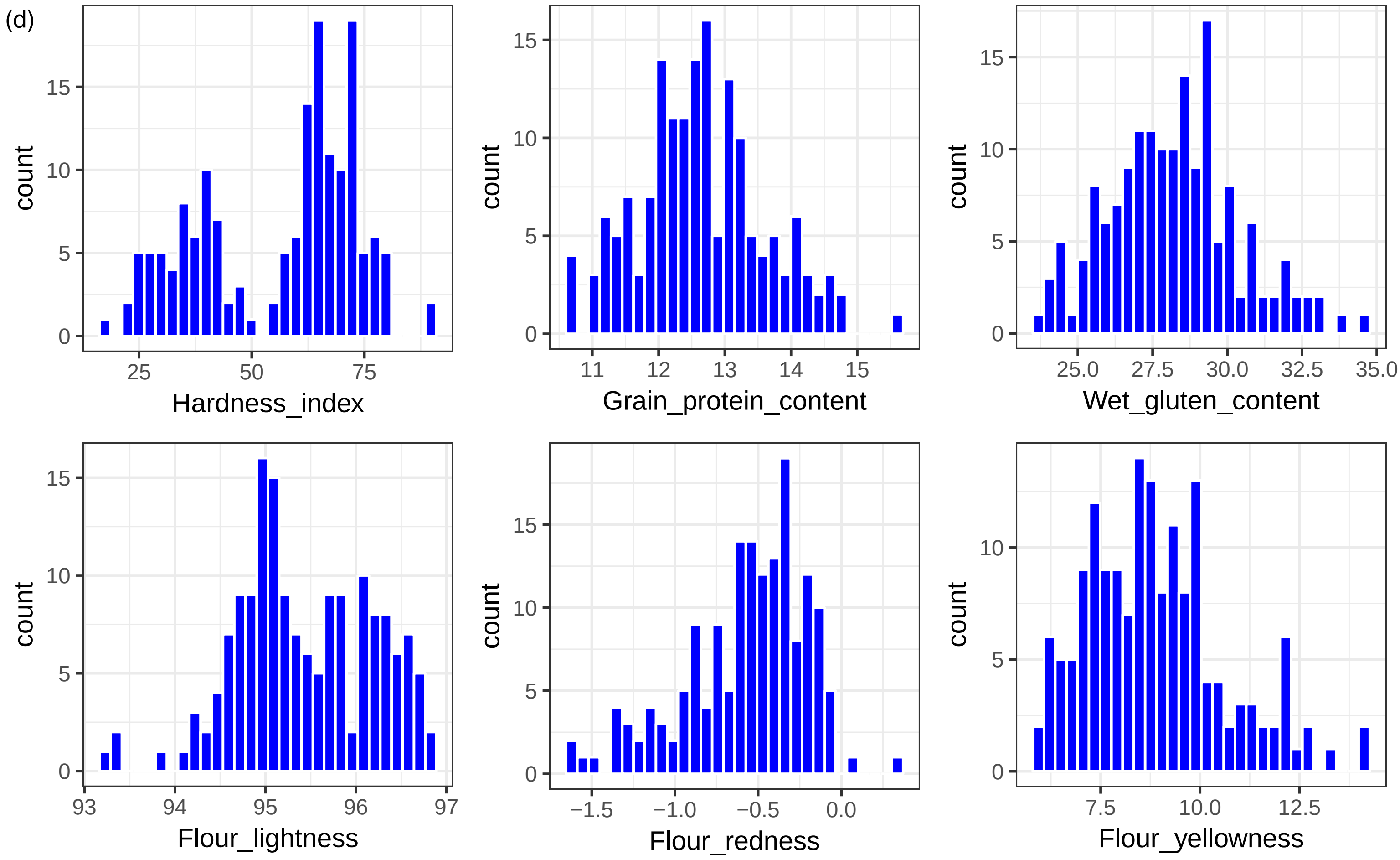

(d)
count
Hardness_index
Grain_protein_content
Wet_gluten_content
Flour_lightness
Flour_redness
Flour_yellowness

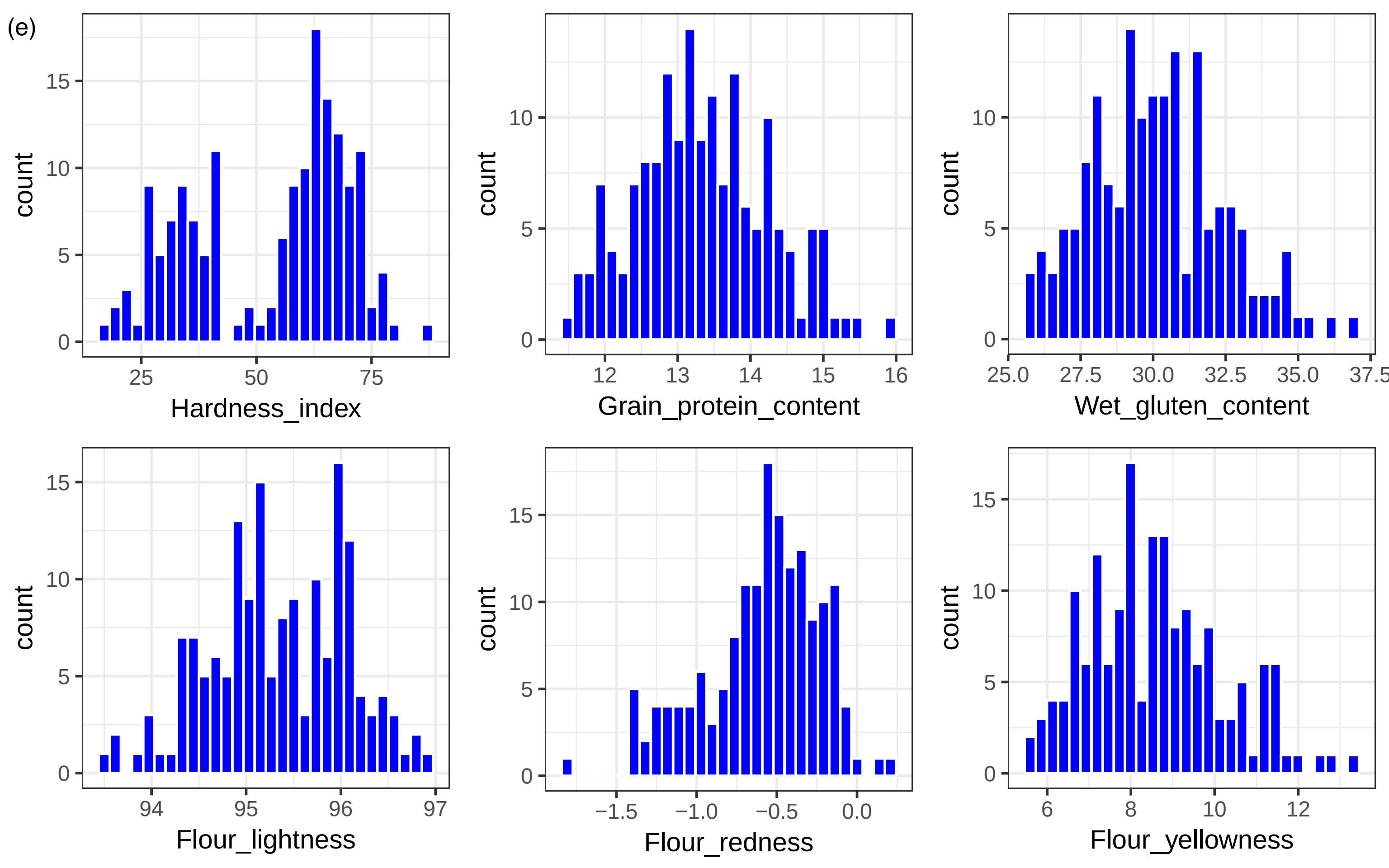
(e)
count
Hardness_index
Grain_protein_content
Wet_gluten_content
Flour_lightness
Flour_redness
Flour_yellowness

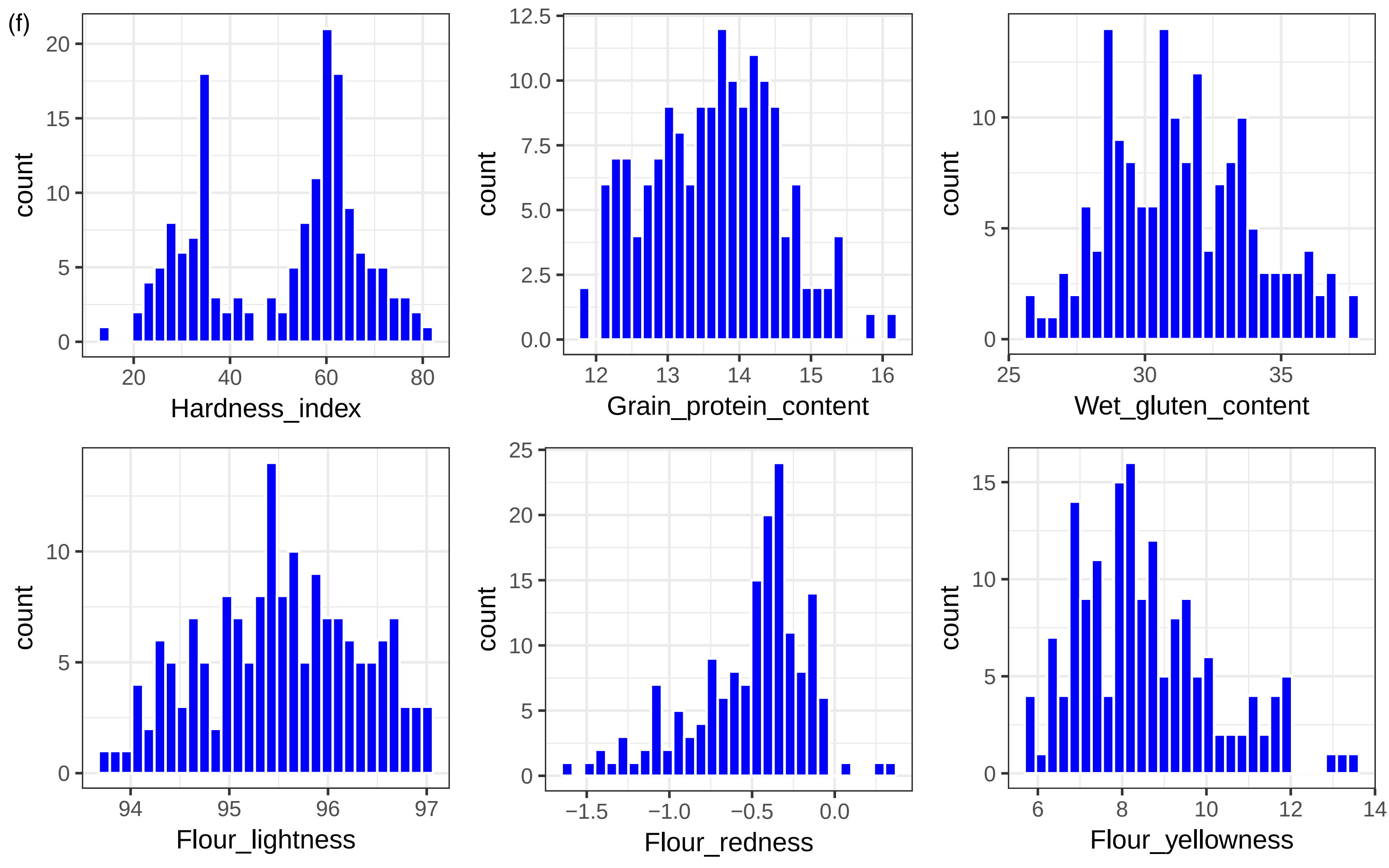

(f)
count
Hardness_index
Grain_protein_content
Wet_gluten_content
Flour_lightness
Flour_redness
Flour_yellowness

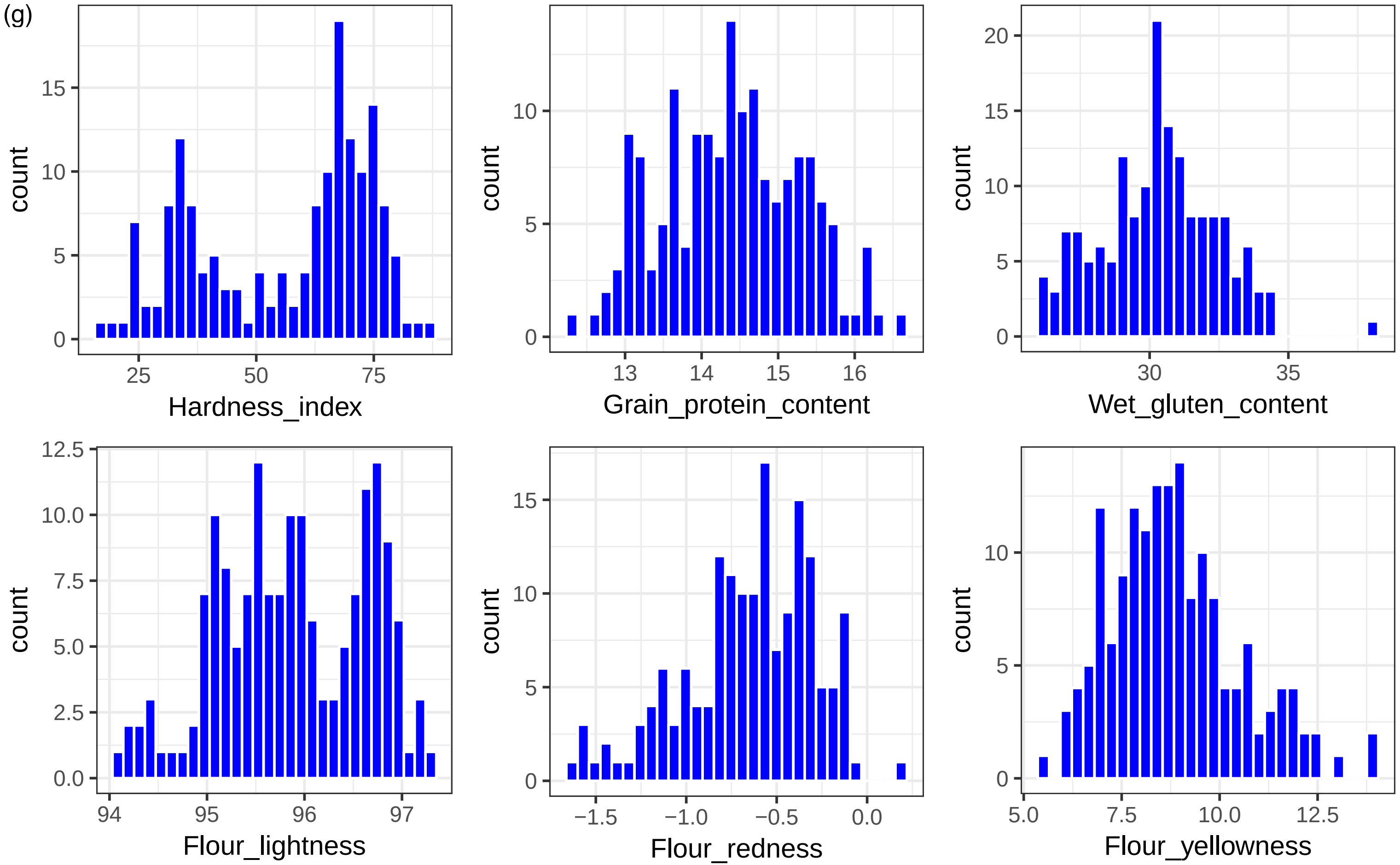
(g)
count
Hardness_index
Grain_protein_content
Wet_gluten_content
Flour_lightness
Flour_redness
Flour_yellowness

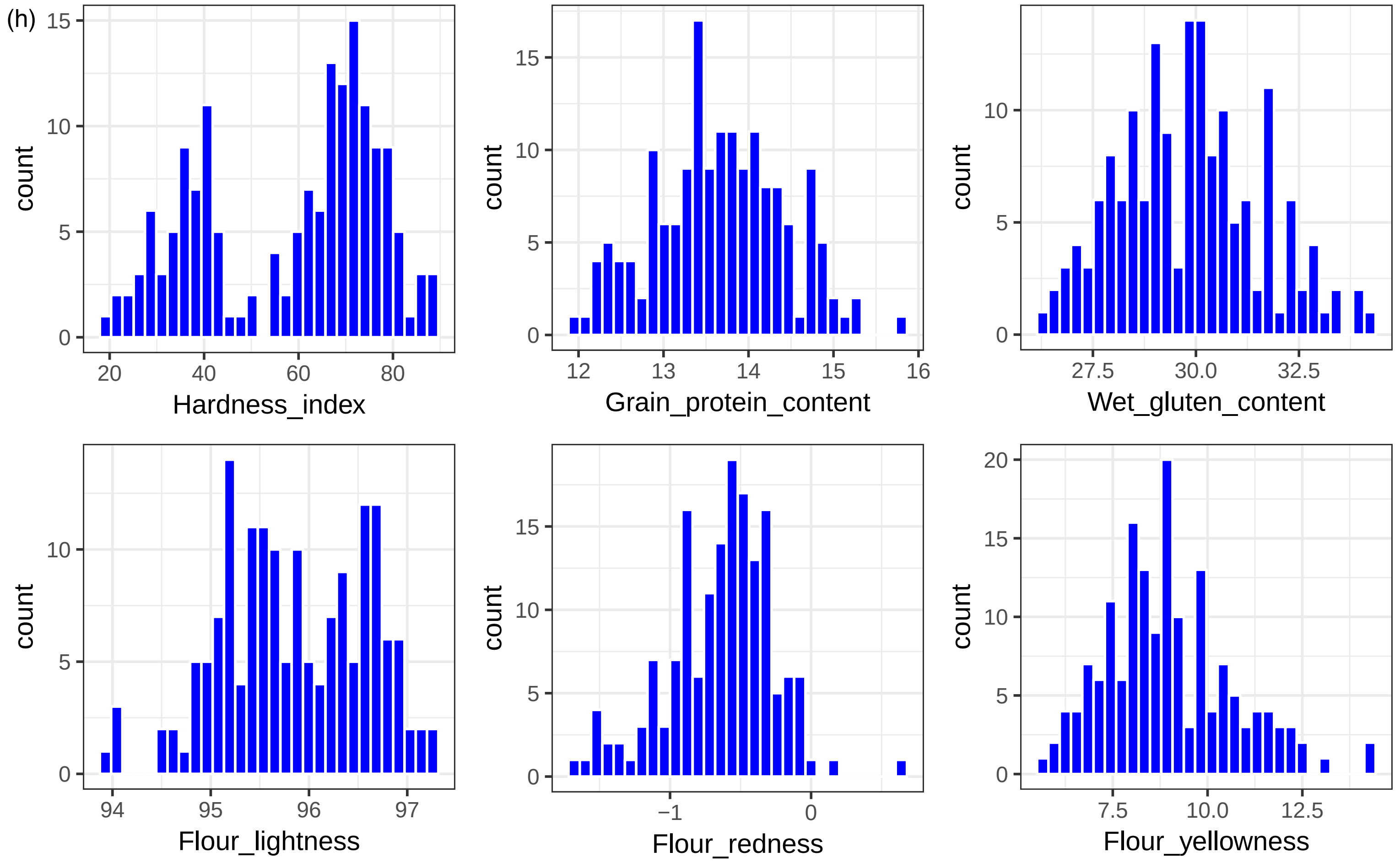
(h)
count
Hardness_index
20
40
60
80
0
5
10
15
Grain_protein_content
12
13
14
15
16
Wet_gluten_content
27.5
30.0
32.5
Flour_lightness
94
95
96
97
Flour_redness
−1
0
Flour_yellowness
7.5
10.0
12.5
20

Fig. S1 Phenotype frequency distribution in eight environments.(a) 2013, Anyang; (b) 2013, Zhengzhou; (c) 2013, Zhumadian; (d) 2014, Anyang; (e) 2014, Zhengzhou; (f) 2014, Zhumadian; (g) 2015, Zhengzhou; (h) 2016, Zhengzhou.
