# Supplementary material for: Genome‐wide association study of six quality traits reveals the association of the TaRPP13L1 gene with flour colour in Chinese bread wheat
Source: Plant Biotechnol J. 2019 Apr 21;17(11):2106–22. doi: 10.1111/pbi.13126 (PMC6790371; doi:10.1111/pbi.13126)
Supplement: Supplementary file 2 — Figure S2 Manhattan and Q–Q plots for HI in 8 environments. [file PBI-17-2106-s009.pdf]

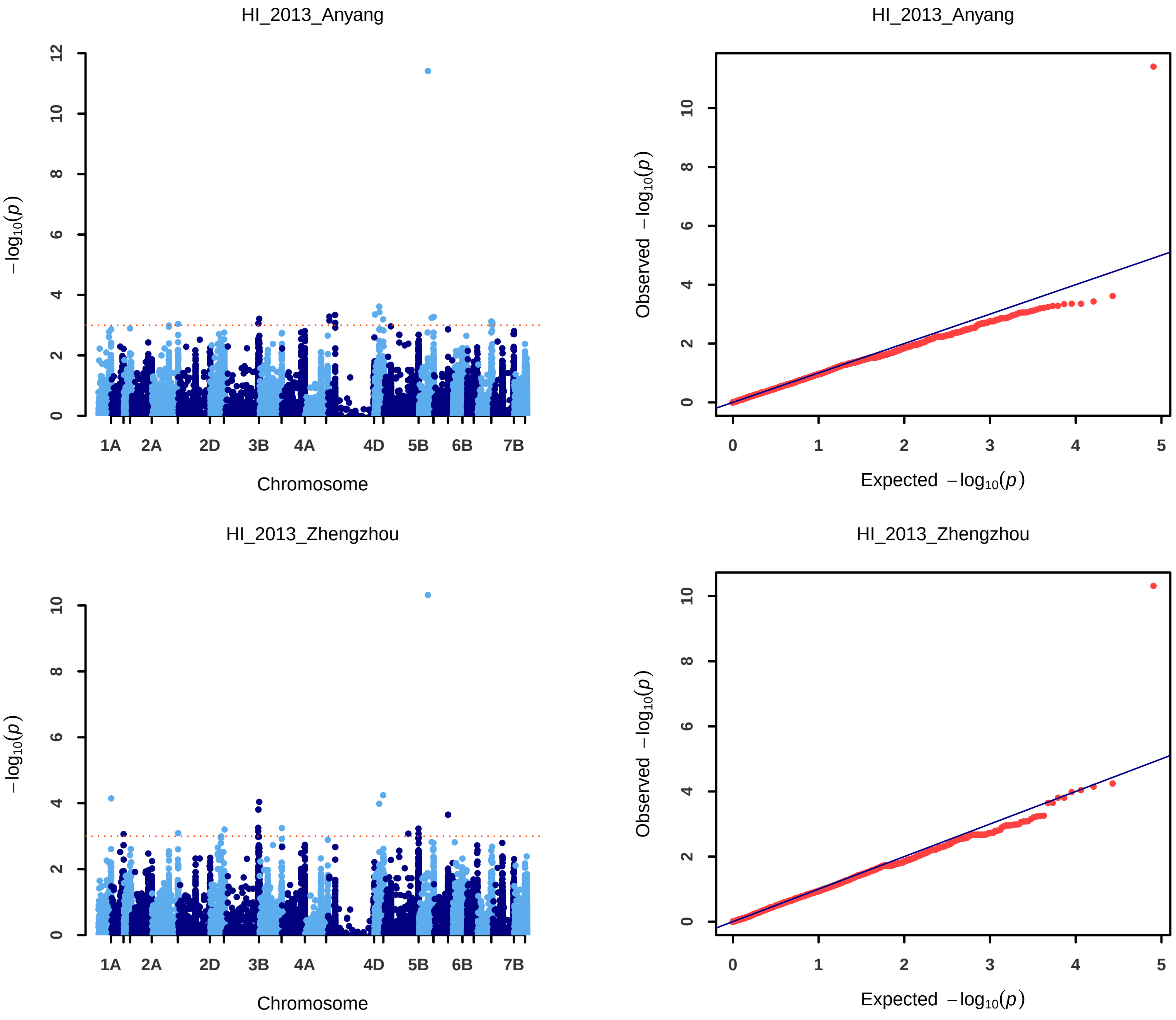
HI_2013_Anyang
−log10(p)
Chromosome
1A 2A 2D 3B 4A 4D 5B 6B 7B
HI_2013_Anyang
Observed −log10(p)
Expected −log10(p)
HI_2013_Zhengzhou
−log10(p)
Chromosome
1A 2A 2D 3B 4A 4D 5B 6B 7B
HI_2013_Zhengzhou
Observed −log10(p)
Expected −log10(p)

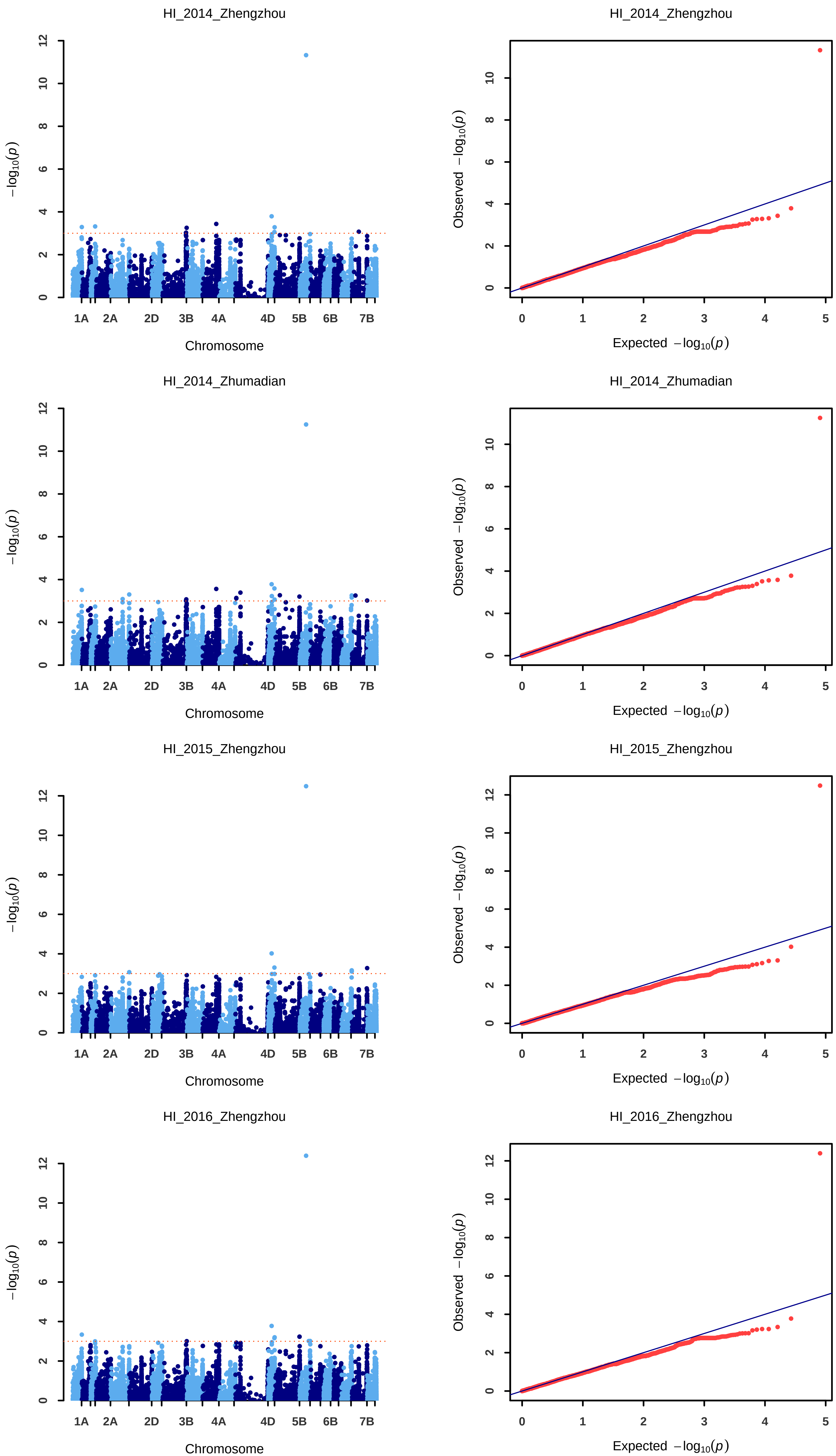
HI_2014_Zhengzhou
HI_2014_Zhumadian
HI_2015_Zhengzhou
HI_2016_Zhengzhou
−log10(p)
1A 2A 2D 3B 4A 4D 5B 6B 7B
Chromosome
Observed −log10(p)
Expected −log10(p)
