# Supplementary material for: Genome‐wide association study of six quality traits reveals the association of the TaRPP13L1 gene with flour colour in Chinese bread wheat
Source: Plant Biotechnol J. 2019 Apr 21;17(11):2106–22. doi: 10.1111/pbi.13126 (PMC6790371; doi:10.1111/pbi.13126)
Supplement: Supplementary file 3 — Figure S3 Manhattan and Q–Q plots for grain protein content in 8 environments. [file PBI-17-2106-s010.pdf]

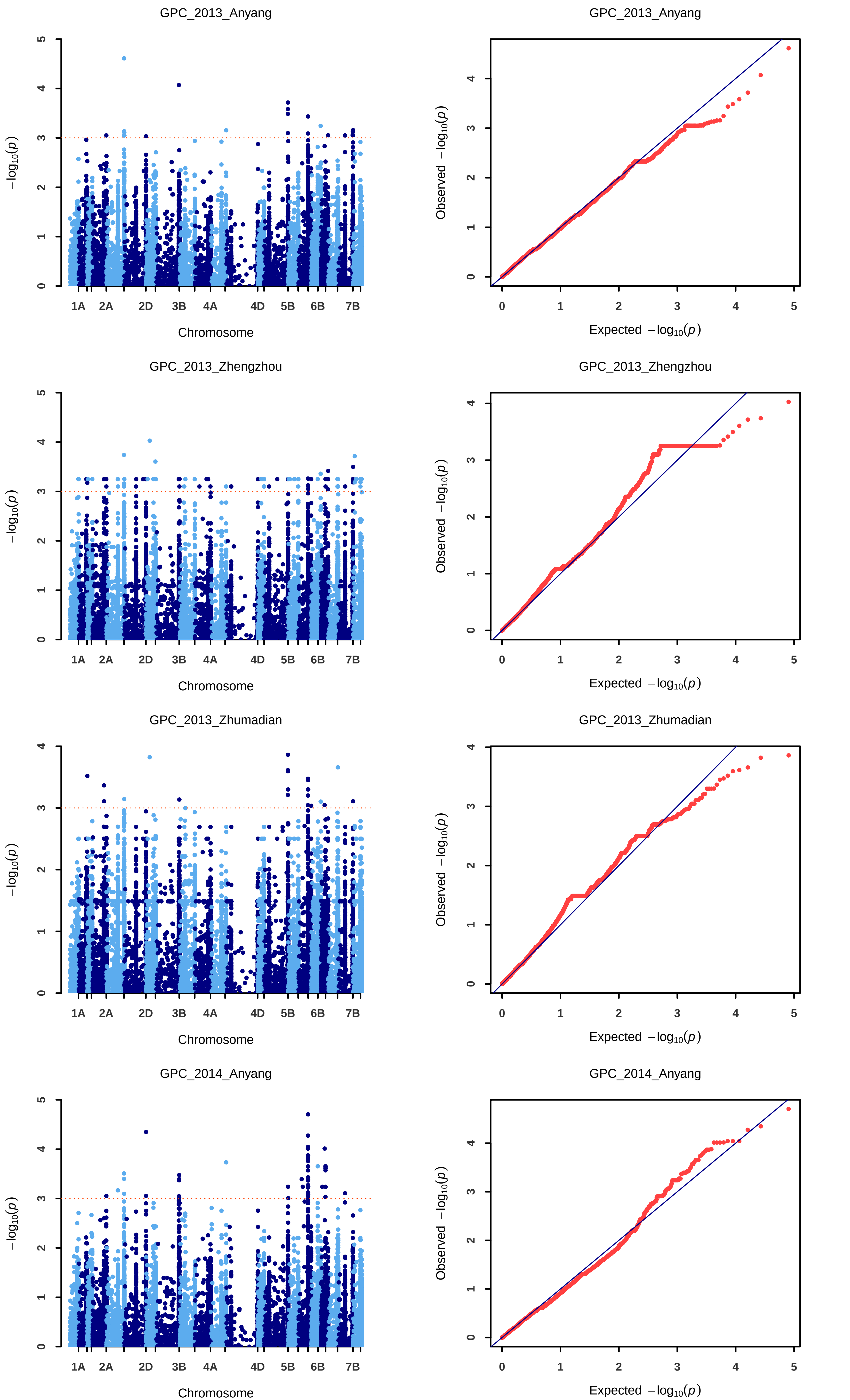
GPC_2013_Anyang
GPC_2013_Zhengzhou
GPC_2013_Zhumadian
GPC_2014_Anyang
$-\log_{10}(p)$
Chromosome
1A 2A 2D 3B 4A 4D 5B 6B 7B
Observed $-\log_{10}(p)$
Expected $-\log_{10}(p)$

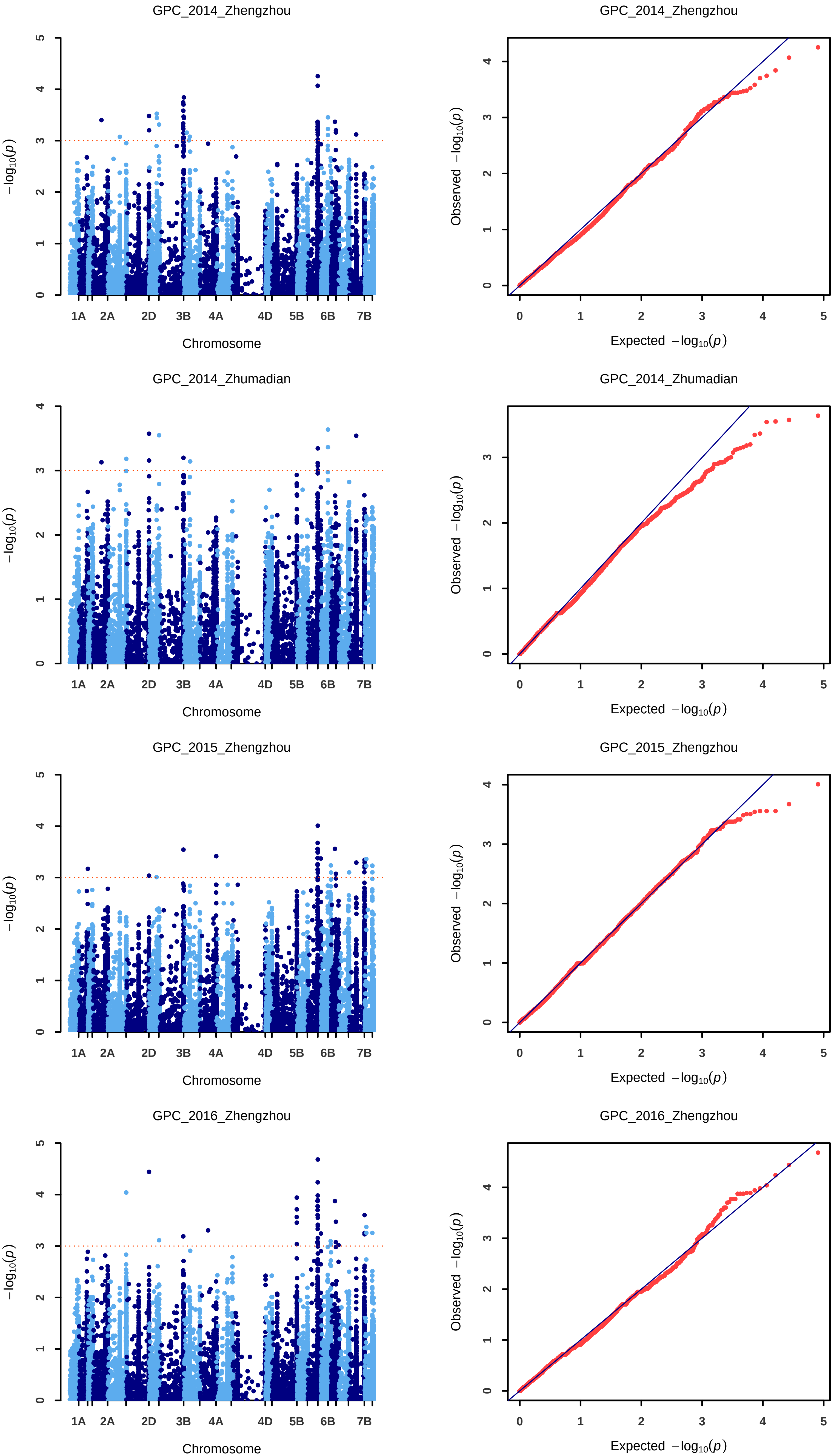
GPC_2014_Zhengzhou
GPC_2014_Zhengzhou
GPC_2014_Zhumadian
GPC_2014_Zhumadian
GPC_2015_Zhengzhou
GPC_2015_Zhengzhou
GPC_2016_Zhengzhou
GPC_2016_Zhengzhou
$-\log_{10}(p)$
1A
2A
2D
3B
4A
4D
5B
6B
7B
Chromosome
Observed $-\log_{10}(p)$
Expected $-\log_{10}(p)$
