# Supplementary material for: Genome‐wide association study of six quality traits reveals the association of the TaRPP13L1 gene with flour colour in Chinese bread wheat
Source: Plant Biotechnol J. 2019 Apr 21;17(11):2106–22. doi: 10.1111/pbi.13126 (PMC6790371; doi:10.1111/pbi.13126)
Supplement: Supplementary file 4 — Figure S4 Manhattan and Q–Q plots for wet gluten content in 8 environments. [file PBI-17-2106-s007.pdf]

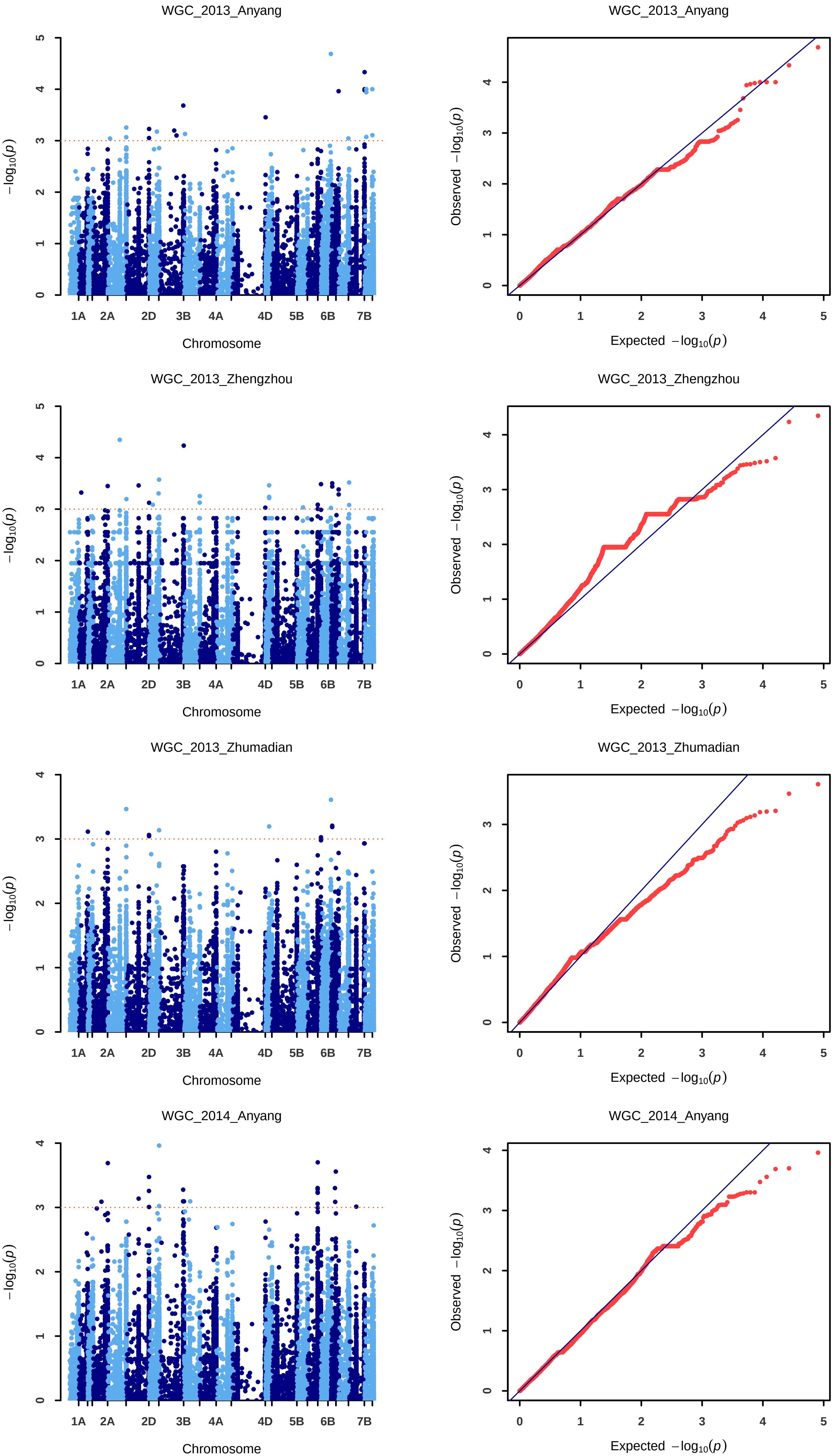

WGC_2013_Anyang
WGC_2013_Zhengzhou
WGC_2013_Zhumadian
WGC_2014_Anyang
$-\log_{10}(p)$
Chromosome
1A 2A 2D 3B 4A 4D 5B 6B 7B
Observed $-\log_{10}(p)$
Expected $-\log_{10}(p)$

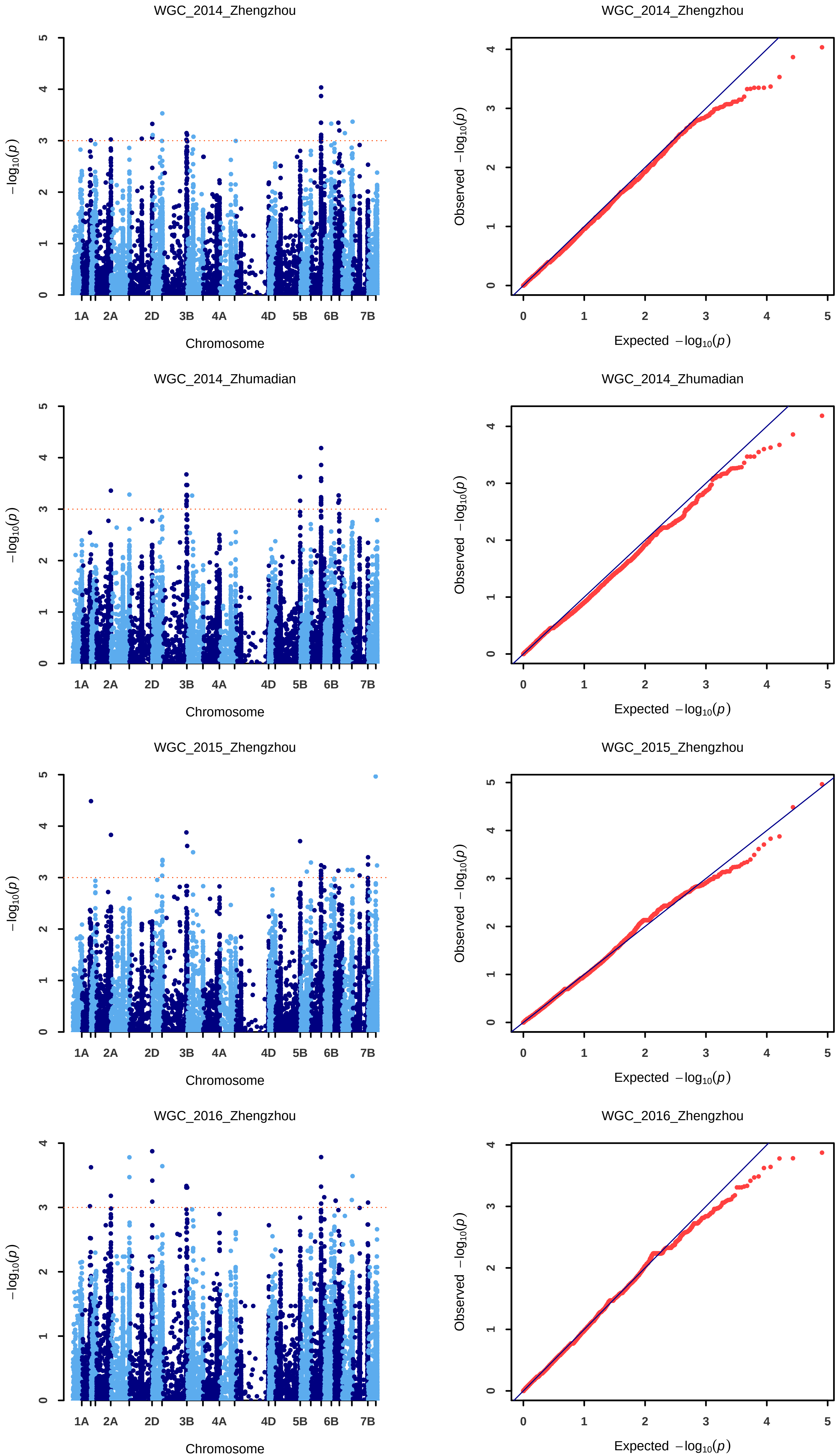
WGC_2014_Zhengzhou
WGC_2014_Zhumadian
WGC_2015_Zhengzhou
WGC_2016_Zhengzhou
$-\log_{10}(p)$
Chromosome
1A 2A 2D 3B 4A 4D 5B 6B 7B
Observed $-\log_{10}(p)$
Expected $-\log_{10}(p)$
