# Supplementary material for: Genome‐wide association study of six quality traits reveals the association of the TaRPP13L1 gene with flour colour in Chinese bread wheat
Source: Plant Biotechnol J. 2019 Apr 21;17(11):2106–22. doi: 10.1111/pbi.13126 (PMC6790371; doi:10.1111/pbi.13126)
Supplement: Supplementary file 7 — Figure S7 Manhattan and Q–Q plots for flour b* in 8 environments. [file PBI-17-2106-s001.pdf]

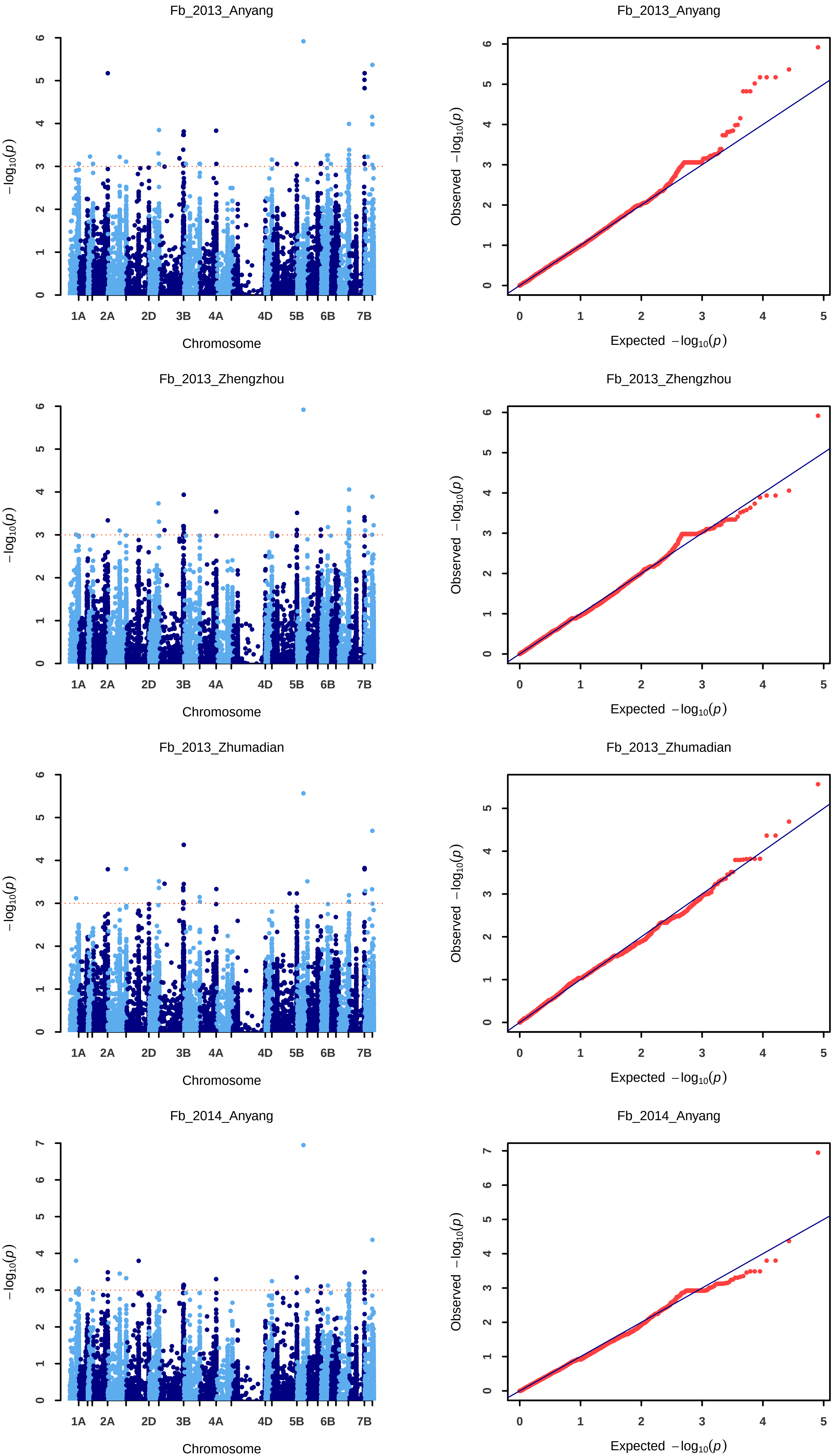

Fb_2013_Anyang
Fb_2013_Anyang
Fb_2013_Zhengzhou
Fb_2013_Zhengzhou
Fb_2013_Zhumadian
Fb_2013_Zhumadian
Fb_2014_Anyang
Fb_2014_Anyang
$-\log_{10}(p)$
Chromosome
1A 2A 2D 3B 4A 4D 5B 6B 7B
Observed $-\log_{10}(p)$
Expected $-\log_{10}(p)$

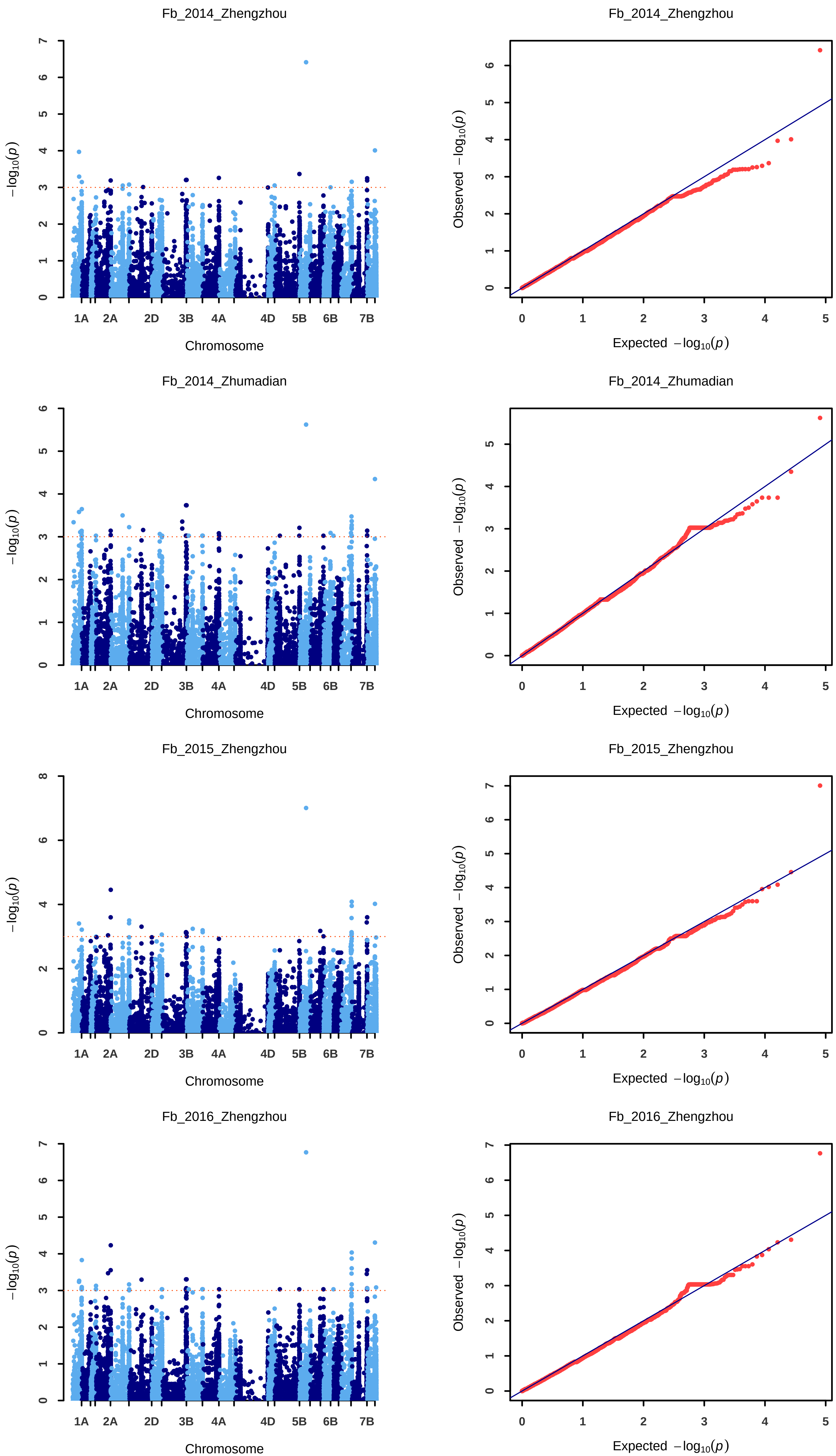
Fb_2014_Zhengzhou
−log10(p)
1A 2A 2D 3B 4A 4D 5B 6B 7B
Chromosome
Fb_2014_Zhengzhou
Observed −log10(p)
Expected −log10(p)
Fb_2014_Zhumadian
−log10(p)
1A 2A 2D 3B 4A 4D 5B 6B 7B
Chromosome
Fb_2014_Zhumadian
Observed −log10(p)
Expected −log10(p)
Fb_2015_Zhengzhou
−log10(p)
1A 2A 2D 3B 4A 4D 5B 6B 7B
Chromosome
Fb_2015_Zhengzhou
Observed −log10(p)
Expected −log10(p)
Fb_2016_Zhengzhou
−log10(p)
1A 2A 2D 3B 4A 4D 5B 6B 7B
Chromosome
Fb_2016_Zhengzhou
Observed −log10(p)
Expected −log10(p)
