# Supplementary figures and images for: Genome‐wide association study of six quality traits reveals the association of the TaRPP13L1 gene with flour colour in Chinese bread wheat
Source: Plant Biotechnol J. 2019 Apr 21;17(11):2106–22. doi: 10.1111/pbi.13126 (PMC6790371; doi:10.1111/pbi.13126)

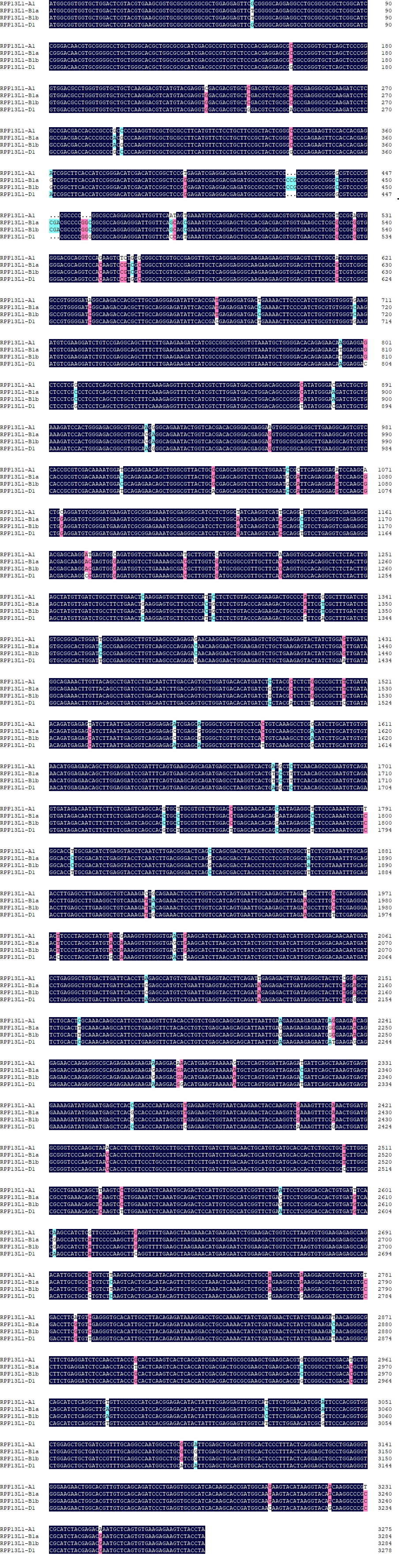

Supplement: Supplementary file 8 — Figure S8 Full alignment of TaRPP13L1 genes and their alleles in different genomes. [file PBI-17-2106-s002.jpg]
